# Artificial Intelligence Governance in Health Systems: Systematic Review of Frameworks and Integrative Model Proposal

**DOI:** 10.2196/87448

**Published:** 2026-06-08

**Authors:** Hassane Alami, Renata Pozelli Sabio, Elsury Johanna Pérez, Marie-Pierre Gagnon, Lyse Langlois, Jean-Louis Denis, Kathy Malas, Lysanne Rivard, Mathilde Savoldelli, Mohamed Ali Ag Ahmed, Jean-Paul Fortin

**Affiliations:** 1 Department of Health Management, Evaluation and Policy, School of Public Health & Center for Public Health Research of the University of Montreal, P.O. Box 6128, Branch Centre-Ville, Montreal, QC, H3C 3J7, Canada, 1 (514) 343-7978; 2 Institute for Data Valorization (IVADO), Montreal, QC, Canada; 3 Faculty of Nursing, Laval University, Quebec, QC, Canada; 4 Department of Industrial Relations, Laval University, Quebec, QC, Canada; 5 Institute of Health Policy, Management and Evaluation, University of Toronto, Toronto, ON, Canada; 6 Integrated Health and Social Services University Network for West-Central Montreal (CIUSSS West-Central Montreal), Montreal, QC, Canada; 7 Territorial Professional Health Community, Lauragais, Occitanie, France; 8 Health Services Management, School of Advanced Public Studies, University of Moncton, Moncton, NB, Canada; 9 Faculty of Medicine, Laval University, Quebec, QC, Canada

**Keywords:** artificial intelligence, digital health, governance, health system, machine learning, deep learning, natural language processing

## Abstract

**Background:**

Several artificial intelligence (AI) governance frameworks have emerged to help health systems (HS) address AI-related risks. However, most fail to capture the multidimensional and evolving nature of real-world governance.

**Objective:**

This systematic review aimed to synthesize existing AI governance frameworks for HS and to propose an integrative AI governance model identifying key components to guide AI-related policy, practice, and research in HS.

**Methods:**

A comprehensive search was conducted in 8 academic databases (PubMed, MEDLINE, Embase, ACM Digital Library, Web of Science, Scopus, Social Sciences Abstracts, and PsycINFO), gray literature databases, and international organization web portals in October 2024 (updates: July 2025 and March 2026) and limited to studies published from November 2014 to March 2026 in English, French, Spanish, or Portuguese. Eligible documents included peer-reviewed articles and reports proposing AI governance frameworks for HS. Two reviewers independently selected the frameworks, assessed their quality using the Appraisal of Guidelines for Research and Evaluation for Health Systems, and extracted data. Results were synthesized using thematic analysis.

**Results:**

The research retrieved 10,175 records, among which 19 AI governance frameworks were identified. Most were published between 2022 and 2024 (n=13, 68%), half (n=10, 53%) were developed by authors based in North America, and only one-third (n=6, 32%) were derived from primary studies. The frameworks focused on 4 levels of AI governance: international (n=3, 16%), national (n=5, 26%), local (n=3, 16%), and organizational (n=8, 42%). All of them underline the crucial role of multidisciplinary bodies in the structure of AI governance in HS. Six key AI governance processes in HS emerged as critical: (1) need and/or problem identification (n=14, 74%), (2) data governance (n=17, 89%), (3) risk assessment and management (n=17, 89%), (4) validation and/or evaluation (n=18, 95%), (5) maintenance and monitoring (n=16, 84%), and (6) integration (n=9, 47%). Additionally, 4 pivotal relational mechanisms were identified: (1) ethical principles and/or values (n=17, 89%), (2) education and training (n=14, 74%), (3) communication (n=12, 63%), and (4) standards and regulations (n=13, 68%).

**Conclusions:**

Our study provides a comprehensive synthesis of existing AI governance frameworks for HS across 4 levels (local, regional, national, and international), underpinned by a quality assessment of the 19 identified frameworks. It differs from existing studies that concentrate on specific dimensions or settings by contributing an integrative AI governance model for HS comprising 2 dimensions and 4 relational mechanisms across the 4 levels, explicitly modeling their interactions. Future research should test and operationalize the proposed model to enhance its practical applicability. Strengthening the methodological rigor of AI governance frameworks will be essential for the responsible integration of AI in HS. As the framework is primarily grounded in Global North and English-language literature, validation in other contexts is warranted.

## Introduction

### Background

The rapid and widespread integration of artificial intelligence (AI) into health systems (HS) has raised high expectations while also creating significant uncertainties and challenges [1-4]. AI refers to “a machine-based system that can, for a given set of human-defined objectives, make predictions, recommendations, or decisions influencing real or virtual environments” [5]. As AI is increasingly viewed as a lever to improve efficiency amidst workforce shortages [6], health organizations are implementing AI tools to support precision diagnostics, personalized medicine, clinical decision-making, and virtual assistance, among other applications [7,8]. The deployment of these complex technologies introduces new pressures and risks, including ethical, sociopolitical, economic, organizational, professional, and clinical challenges, with particular sensitivities around patient safety and privacy [1,3,4,9]. Additional concerns include bias and inequities across subgroups, regulatory uncertainty, liability, vendor lock-in, technical debt, data quality and interoperability gaps, inadequate management and training, and misalignment between procurement incentives and demonstrated real-world value [10,11]. In response, stakeholders have called for robust and adaptive AI governance frameworks for HS to safeguard the system and enable responsible, sustainable, and effective integration of AI-based technologies [1,12-15].

Although several ethical AI frameworks for HS define fundamental values (eg, trust, transparency, accountability, safety, justice, fairness, nonmaleficence, and privacy) and normative objectives [16,17], they generally do not provide benchmarks or operational guidance for achieving them [17-19]. While the role of ethical frameworks is to articulate principles, the role of governance frameworks is to provide the operational structures, processes, and mechanisms (the “how”) required to translate principles (the “what”) into practice [20-24]. To this end, governance frameworks identify relevant actors, allocate decision-making authority, and clarify responsibilities [20,21,23-25]. They also guide core functions such as data governance, model validation and auditing, and risk assessment and management [20,21,23-25]. To support a practical and consistent application of AI ethical principles [23], governance frameworks should aim to operationalize the following: (1) roles, rules, norms, competencies, and policies applicable throughout the AI lifecycle; (2) value-sharing arrangements, procurement and contracting requirements, remuneration and reimbursement mechanisms, and intellectual property considerations; (3) auditable requirements regarding data governance, human oversight, explainability, model validation, and cybersecurity; (4) postdeployment monitoring and incident reporting; assessments of clinical effectiveness, safety, cost implications, and equity impacts; and liability and risk allocation; and (5) stakeholder engagement, transparent public communication, accountability mechanisms, and criteria for model replacement or retirement [23,26-32].

Although AI governance in HS presents distinct characteristics and requirements, it nonetheless forms an integral component of the broader governance architecture for digital health [1,12,25,33-35]. It is grounded in the institutional, regulatory, and infrastructural arrangements and structures that shape how digital technologies are designed, procured, deployed, used, evaluated, and monitored in real-world care settings and that ultimately determine whether these tools can be integrated safely, effectively, efficiently, sustainably, and equitably at scale [1,12,25,34]. Accordingly, we adopt a governance perspective consistent with digital health governance and define AI governance in HS as “the exercise of political, administrative, and technical authority to manage everything associated with the health information system, in all areas of a national system. The structure of this governance consists of the mechanisms, processes, and institutions through which all stakeholders articulate their interest, exercise their rights, meet their obligations, and resolve their differences” [33]. These governance elements are interconnected, forming a coherent whole that ensures AI-based technologies meet clinical, legal, ethical, and regulatory requirements and are used in alignment with HS strategies, objectives, principles, and values [35].

The pressing need for comprehensive AI governance frameworks in HS has led to a proliferation of proposals over the past decade. Yet, these initiatives often address sociopolitical, ethical, clinical, or data management issues separately, rather than treating them as interdependent dimensions of governance [36]. This literature remains limited by 2 persistent conceptual shortcomings: the absence of a shared definition of AI governance in HS and the frequent conflation of governance with related notions such as ethics, responsibility, or regulation. For example, a scoping review by Stogiannos et al [37] mapped the available literature on AI governance in medical imaging and radiology and proposed a governance framework. However, the authors did not clearly define what they meant by governance; their analysis primarily examined the factors relevant to governance in the United Kingdom’s imaging and radiology sectors, and most of the documents reviewed emphasized ethical and regulatory considerations at the organizational level rather than governance in a broader institutional sense [37]. This fragmentation, combined with a tendency to underestimate the structural and operational demands of governance, has contributed to persistent misalignment between national ambitions for AI integration and the strategies, institutional arrangements, and resources actually deployed to support such integration [36].

### Objective

To the best of our knowledge, no systematic review has yet identified and synthesized AI governance frameworks in HS, despite the fact that such analysis is essential for identifying recurrent shortcomings and informing strategies for the responsible integration of AI-based technologies into HS [38]. This systematic review aimed to synthesize existing AI governance frameworks for HS and to propose an integrative AI governance model identifying key components to guide AI-related policy, practice, and research in HS.

## Methods

### Overview

This systematic review was conducted and reported in accordance with the PRISMA (Preferred Reporting Items for Systematic Review and Meta-Analyses) Literature Search Extension statement (Checklist 1) [39,40]. The full description of methods is provided in the protocol registered on the Open Science Framework prior to data collection [41]. No modifications or additional information were made to the protocol following its registration (PRISMA item 24c). In addition, the following PRISMA checklist items were considered not applicable to this review: 10b, 12, 13e, 13f, 15, 19, 20b, 20d, and 22 (Checklist 1). Given the qualitative nature of this systematic review, these items were not relevant to the methodological approach adopted in this study. Ethical approval was not required for this systematic review.

### Eligibility Criteria

The evidence map included published peer-reviewed articles or reports proposing a new framework, guideline, standard, or position statement of AI governance in HS. Eligible documents had to be explicitly labeled as AI governance in HS by the authors. Only records published in English, French, Spanish, or Portuguese between November 2014 and March 2026 were included. We selected 2014 as the starting point because the mid-2010s marks the period when AI began transitioning from primarily experimental applications to more concrete and integrated uses within HS [42-44]. This period also coincides with the expansion of large-scale digital health data infrastructures and the growing involvement of major technology companies in health AI ecosystems. From a governance perspective, it corresponds to the emergence of more structured global discussions on AI ethics, responsible AI, and digital health governance, as well as the proliferation of national digital health and AI strategies [43,44]. We therefore chose 2014 to focus the analysis on frameworks most relevant to current policy and implementation contexts. Abstracts, letters to the editor, commentaries, essays, viewpoints, conference proceedings, and unavailable articles were excluded. Two reviewers (RPS and EJP) independently screened all references for inclusion using *Covidence* (a web-based software platform) [45]. Disagreements were resolved through discussion. If the 2 primary reviewers could not reach a consensus, a third reviewer (HA) adjudicated the final decision.

### Information Sources and Search Strategy

A comprehensive literature search was conducted in 8 databases (PubMed, MEDLINE [Ovid], Embase [Ovid], ACM Digital Library, Web of Science [Clarivate], Scopus, Social Sciences Abstracts [EBSCO], and PsycINFO [EBSCO]) in October 2024 (updated in July 2025 and March 2026). Database searches were conducted and limited to human studies published since November 2014. The strategy combined free-text keywords and controlled vocabulary terms structured around the concepts of AI (“Artificial Intelligence” or “AI” or “machine learning” or “deep learning” or “natural language processing”) AND governance (“governance” or “regulation” or “framework” or “model” or “policy” or “guideline” or “ethic” or “standard” or “principles” or “regulatory” or “responsible”) AND health system (“health system” or “healthcare system” or “health services” or “health sector” or “hospital” or “healthcare delivery” or “public health systems” or “health sector”). The search strategy was developed by the authors through a rapid literature review and group discussion. We conducted searches across Google Scholar, ProQuest Dissertations and Theses Database (ProQuest), Social Science Research Network (SSRN eLibrary), and Evidence for Informed Health Policymaking (PDQ-Evidence). The electronic search was complemented by manual searches of the reference lists of relevant articles and web portals of institutions including the World Health Organization (WHO), United Nations, the Organisation for Economic Co-operation and Development, World Bank, European Union, National Institute for Health and Care Excellence, and National Institutes of Health. Details on the search strategies are available in Multimedia Appendix 1. All references retrieved from the searches were imported into *EndNote20* (Clarivate) to manage citations and remove duplicates. The refined list of citations was imported into *Covidence*.

### Data Collection Process

Data were extracted using a standardized form in *Microsoft Excel,* including (1) document characteristics (author names, year of publication, first author country, objective, type of document, targeted audience, and definition of AI governance); (2) methodology (research question, study design, sample characteristics, and data collection procedures); (3) framework characteristics (name, level of application, design, actors, roles and accountabilities, governance structure functions, processes, mechanisms, barriers, and challenges); and (4) funding sources. Data were extracted by 2 reviewers (RPS and LR) and checked by 2 others (EJP and HA).

### Selection Process: Study Quality Appraisal Using AGREE-HS

Framework quality was assessed using a modified version of the Appraisal of Guidelines for Research and Evaluation for HS (AGREE-HS). The original AGREE-HS and the modifications are presented in Multimedia Appendix 2 [46]. As in the original AGREE-HS, 5 quality items were rated: (1) topic (clarity and relevance of the AI governance challenge); (2) participants (stakeholder inclusion, multidisciplinary representation, and conflicts of interest); (3) methods (transparency and rigor of framework development processes); (4) recommendations (clarity, feasibility, ethical principles, equity, and sociocultural and political factors); and (5) implementability (feasibility, barriers and facilitators, resource requirements, sustainability, and transferability). Regarding modifications, 21 criteria of the original AGREE-HS applicable to frameworks were also included, as no tool currently exists for these.

Additionally, we assigned scores to each criterion in advance to improve assessment reliability. Two authors (RPS and EJP) independently applied the explicit scoring criteria to each item using a Microsoft Excel spreadsheet. Disagreements in each item score were resolved by consensus. Each domain score was calculated by aggregating its items' scores and was rated on a 7-point scale, where 1 indicated the lowest quality and 7 indicated the highest quality. The framework overall score was calculated following the AGREE-HS manual as follows [47]: (obtained score–minimum score)/(maximum score–minimum score)×100%, with a minimum total score of 5 and a maximum total score of 35. We performed a tertile split of the frameworks’ overall scores using the 33rd- and 67th-percentile cut points to classify them as high quality (overall scores >51%), moderate quality (overall scores between >41% and 51%), or low quality (overall scores ≤41%).

### Data Synthesis

Data were analyzed and integrated through a narrative synthesis approach, which allowed for a coherent combination of the varied elements within the AI governance frameworks for HS. Framework levels were categorized as international, national, local, or organizational based on their scope. The analysis was informed by the framework of Van Grembergen et al [48] for IT governance adapted to AI-based technologies. This framework encompasses 3 interconnected dimensions of governance: structures, processes, and relational mechanisms. The structures are the actors and their roles that ensure a clear chain of responsibility for the decisions and operations around AI-based technologies. The processes are the formal methods and workflows for making and monitoring these technologies, including establishing a framework for planning, prioritizing, and monitoring AI initiatives. Relational mechanisms are elements that foster trust, participation, partnership, and mutual understanding in the processes [48]. Whenever it is possible and pertinent, the components of the processes and relational mechanisms identified in the frameworks are presented according to the different stages of the AI lifecycle.

## Results

### Framework Selection

**Figure 1.**
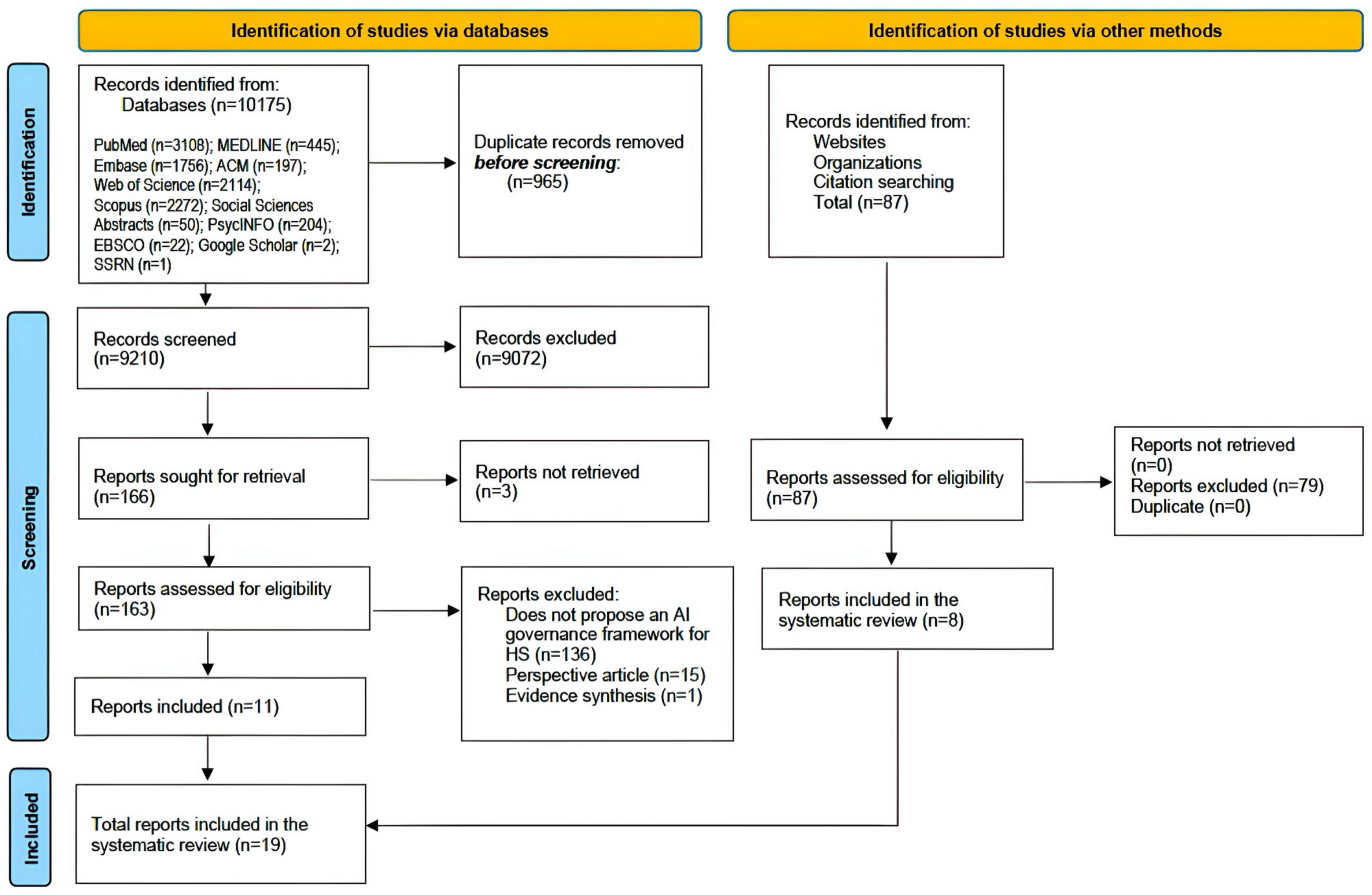
PRISMA (Preferred Reporting Items for Systematic Reviews and Meta-Analyses) for literature screening [49]. AI: artificial intelligence; HS: health systems.

A total of 10,175 records were retrieved from the bibliographic databases, of which 91% (n=9210) remained after removing duplicates. Following title and abstract screening, 163 (2%) documents were full-text screened. Of these, 12 (0.1%) articles proposed an AI governance framework for HS. One (0.009%) article was excluded, as it was a scoping review [37], resulting in the inclusion of 11 (0.1%) peer-reviewed articles. Another 87 additional full-text records were identified through websites and reference lists, adding 8 publications to the systematic review. A total of 19 AI governance frameworks for HS were identified in 15 peer-reviewed articles and 4 reports (Figure 1).

### Framework Characteristics

**Figure 2.**
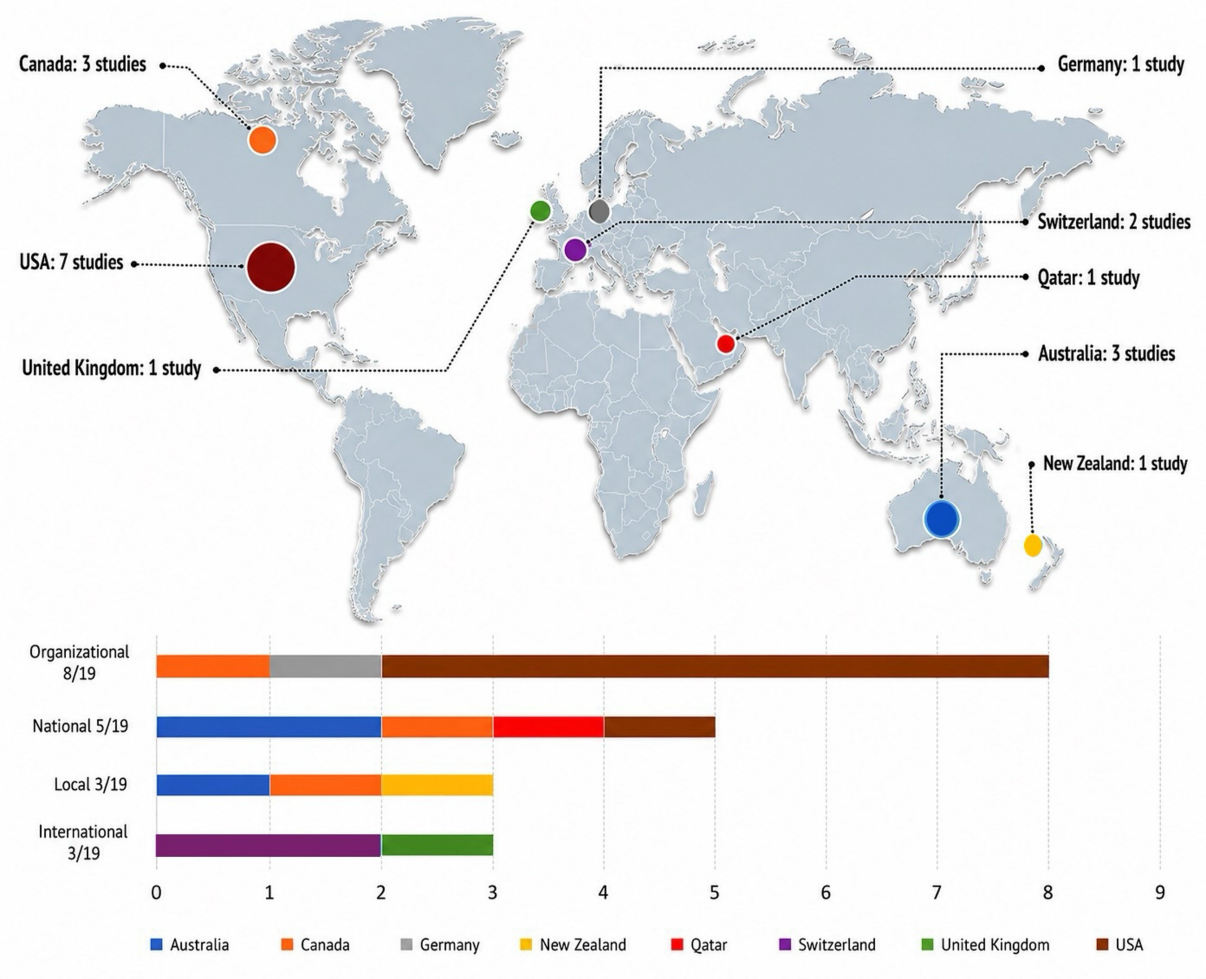
Geographical distribution of first authors and level of intended audience (organizational, local, national, and international).

An overview of included documents is presented in Multimedia Appendix 3. Six documents resulted from primary studies [50-55], and only 6 provided a definition of AI governance [1,56-60]. The geographical distribution of first authors and the scale of intended audiences are presented in Figure 2. Most frameworks (13/19, 68%) were published between 2022 and 2024 [50-57,59,61-64]. Ten (53%) frameworks were developed by authors based in North America—7 (37%) from the USA [50,55,57,59-61,63] and 3 (16%) from Canada [58,62,65]—3 (16%) in Australia [30,53,64], 2 (10%) in Switzerland [1,56], 1 (5%) in the United Kingdom [51], 1 (5%) in Germany [52], 1 (5%) in New Zealand [54], and 1 (5%) in Qatar [66]. Details of the included frameworks are presented in Multimedia Appendix 4. They targeted audiences at 4 levels: 3 international [1,51,56], 5 national [30,59,64-66], 3 local [53,54,62], and 8 organizational [50,52,55,57,58,60,61,63]. Ten frameworks were developed through collaboration between academics and practitioners [1,50,55,56,59,60,63-66].

### Framework Critical Appraisal

Table 1 presents the results of the adapted AGREE-HS. Seven frameworks were rated as high quality [1,30,50,51,56,60,62], 6 were rated as moderate quality [53,54,58,61,63,66], and 6 were rated as low quality [52,55,57,59,64,65].

While 9 frameworks received a score of 6 or higher for the information about the framework development team (Table 1—Participants) [50,51,53,54,56,61-63,65], only 7 reached this threshold for describing the AI governance challenge, its causes, and the priority attributed to it (Table 1—Topic) [1,51,56,58,60,62,66]. Most of the authors do not provide a complete description of systematic and transparent methods to review the evidence and to agree upon the framework components. Therefore, 14 frameworks scored 3 or lower on the dimension assessing the methods used to develop them (Table 1—Methods) [1,30,50,52,53,55,57,59,61-66]. Finally, most authors did not include information on the framework implementability (17 scored 3 or less; Table 1—Implementability) [1,30,51-56,58-66].

**Table 1.** Results of the Appraisal of Guidelines for Research and Evaluation for Health Systems (AGREE-HS) and classification of the frameworks according to their quality^a^.

References	Participants	Topic	Methods	Recommendations	Implementability	Overall score (%)	Quality
International level							
WHO^b^, 2024 [56]	7	7	6	6	2	77	High
Morley et al, 2022 [51]	6	7	7	5	2	73	High
WHO, 2021 [1]	4	7	3	5	3	57	High
National level							
Reddy et al, 2020 [30]	5	5	3	5	3	53	High
Jaremko et al, 2019 [65]	6	5	2	2	1	37	Low
Parker et al, 2024 [59]	4	3	3	4	2	37	Low
AAAiH^c^, 2023 [64]	4	4	2	3	1	30	Low
Solaiman 2025 [66]	4	7	1	5	3	50	Moderate
Local level							
Arnaout et al, 2024 [62]	7	7	2	4	1	53	High
Whittaker et al, 2023 [54]	6	4	4	4	1	47	Moderate
Carter et al, 2024 [53]	7	2	3	5	1	43	Moderate
Organizational level							
Liao et al, 2022 [50]	6	4	2	3	6	53	High
Bedoya et al, 2022 [61]	7	5	2	3	2	47	Moderate
Hassan et al, 2025 [58]	3	6	5	4	1	47	Moderate
Economou-Zavlanos et al, 2024 [63]	6	3	3	3	3	43	Moderate
Daye et al, 2022 [57]	4	4	3	2	4	40	Low
Kim et al, 2023 [55]	4	4	3	4	1	37	Low
Apfelbacher et al, 2024 [52]	2	3	2	1	1	31	Low
Kim et al, 2026 [60]	5	7	5	4	3	67	High

a Score ranging from 1 to 7: score of 1 (lowest quality): a score of 1 should be given if there is no information that is relevant to the AGREE-HS item, if the criteria are very poorly reported in the HSG document or if the authors explicitly state that it was not done. Score of 7 (highest quality): a score of 7 should be given if the information related to the AGREE-HS item was exceptionally well reported, all criteria related to the item have been considered during the development of the guidance, and the information related to the item is applicable in its context. The frameworks were classified into high quality (overall scores >51%), low quality (overall scores ≤41%), and moderate quality (overall scores between >41% and 51%). The details about the evaluation components can be found in Multimedia Appendix 2.

b WHO: World Health Organization.

c AAAiH: Australian Alliance for Artificial Intelligence in Healthcare.

### Governance Structures

AI governance structures encompass functions and the roles and responsibilities of involved actors. Multimedia Appendix 5 presents authors’ statements on the functions of AI governance structures. Seventy-four percent (14/19) of frameworks explicitly define these functions [1,30,50,51,53,54,56,59-63,65,66]. Two international-level frameworks emphasize that these structures are intended to support the achievement of national health objectives [1,51].

According to Morley et al [51] and the WHO [1], AI governance structures also play a crucial role in establishing shared standards of safety, privacy, and efficacy to ensure compliance with minimum requirements. Furthermore, the WHO indicates that these structures can help prevent regulations that could create unfair competitive advantages or disadvantages for both companies and governments. Such structures also ensure that governments develop regulatory frameworks that uphold ethical principles, human rights, and international law [56].

At a national level, 4 frameworks indicate that AI governance structures have the function of establishing guidelines to evaluate AI-based technologies intended for use in the HS [30,59,65,66]. According to Reddy et al [30], Parker et al [59], and Solaiman [66], these guidelines help to ensure that AI-based technologies are implemented safely, ethically, and in compliance with regulatory standards. Likewise, for 3 local-level frameworks, the function of AI governance structures is to ensure adherence to ethical principles in the deployment of AI, such as equity, privacy, and transparency [53,54,62]. Arnaout et al [62] and Whittaker et al [54] highlighted that these structures also play a crucial role in identifying gaps for AI solutions and ensuring the appropriateness, safety, and effectiveness of AI development and deployment. Additionally, 6 organizational-level frameworks underscore the pivotal role of AI governance structures in providing guidance and support throughout the AI lifecycle [50,57,58,60,61,63]. For some, these structures are responsible for supporting the development of AI-based technologies [57,58,60] and for establishing evaluation, monitoring, and adoption processes [50,57,58,60].

**Figure 3.**
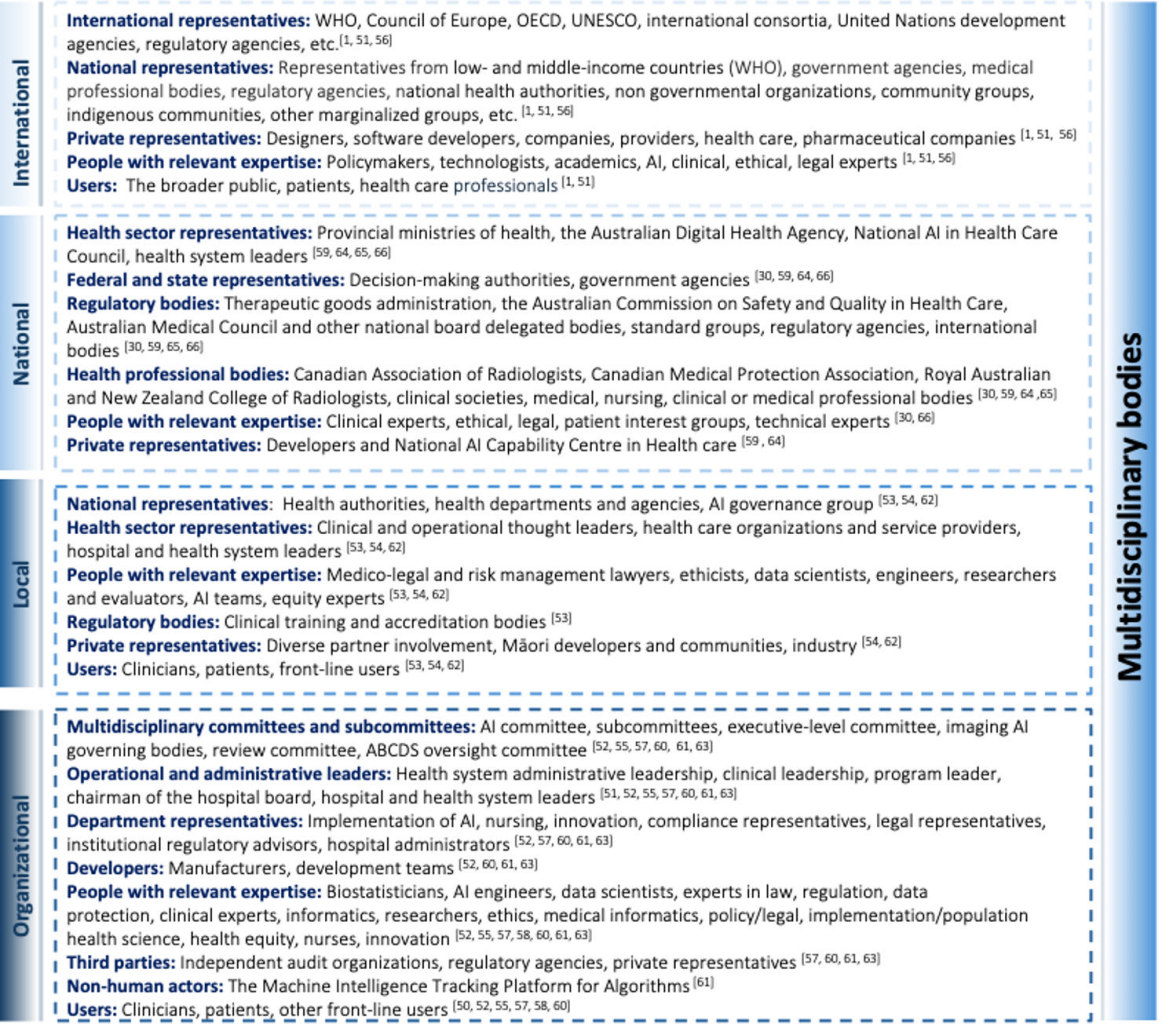
Artificial intelligence (AI) governance actors identified across the included frameworks. OECD: Organisation for Economic Co-operation and Development; UNESCO: United Nations Educational, Scientific and Cultural Organization; WHO: World Health Organization.

The literature on actors and their roles and responsibilities is notably more heterogeneous. Across the 19 frameworks, actor composition within governance structures at all levels varied substantially, reflecting differences in contextual scope and sectoral representation. These actors are categorized by subgroup and scale in Figure 3; however, the categories overlap. All frameworks underlined the crucial role of multidisciplinary bodies or teams in governing AI within HS across these different levels. Notably, only the international-level frameworks explicitly highlight the importance of synergy between governance structures operating at different scales [1,51,56].

Owing to the large number of actors involved, the frameworks present significant variations in the roles and responsibilities assigned to each actor, with many responsibilities shared among multiple actors. For instance, while Morley et al [51] suggested that “collaboration and multidisciplinary working by decision-makers, technologists, healthcare professionals, and academics are needed to ensure appropriate expertise throughout the AI life cycle,” the WHO [56] stated that “governments could require developers to ensure that the design and development of a general-purpose foundation model achieve certain outcomes throughout its life cycle.” For this reason, it was not feasible to synthesize the roles and responsibilities comprehensively. Other examples of actors’ roles and responsibilities are provided in Multimedia Appendix 6.

### Governance Processes

#### Overview

Governance processes involve the formal methods and workflows for developing and monitoring AI-based technologies. Within the included frameworks, 6 processes were identified as critical: need and/or problem identification, data governance, risk assessment and management, validation and/or evaluation, maintenance and monitoring, and integration. Table 2 presents the frameworks that included these processes as crucial in AI governance within HS.

**Table 2.** Artificial intelligence governance processes identified across the included frameworks.

Author and year	Need and/or problem identification^a^	Data governance^b^	Risk assessment and management^c^	Validation and/or evaluation^d^	Maintenance and monitoring^e^	Integration^f^
International level						
WHO^g^, 2024 [56]	X	X	X	X		
Morley et al, 2022 [51]	X			X	X	
WHO, 2021 [1]	X	X	X	X	X	
National level						
Reddy et al, 2020 [30]		X	X	X	X	X
Jaremko et al, 2019 [65]		X				
Parker et al, 2024 [59]	X	X	X	X	X	
AAAiH^h^, 2023 [64]		X	X	X	X	
Solaiman, 2025 [66]		X	X	X	X	X
Local level						
Arnaout et al, 2024 [62]	X	X	X	X	X	
Whittaker et al, 2023 [54]	X	X	X	X	X	X
Carter et al, 2024 [53]	X	X	X	X	X	
Organizational level						
Liao et al, 2022 [50]			X	X	X	
Bedoya et al, 2022 [61]	X	X	X	X	X	X
Hassan et al, 2025 [58]	X	X	X	X		X
Economou-Zavlanos et al, 2024 [63]	X	X	X	X	X	X
Daye et al, 2022 [57]	X	X	X	X	X	
Kim et al, 2023 [55]	X	X	X	X	X	X
Apfelbacher et al, 2024 [52]	X	X	X	X	X	X
Kim et al, 2026 [60]	X	X	X	X	X	X

a Total frameworks=74%.

b Total frameworks=89%.

c Total frameworks=89%.

d Total frameworks=95%.

e Total frameworks=84%.

f Total frameworks=47%.

g WHO: World Health Organization.

h AAAiH: Australian Alliance for Artificial Intelligence in Healthcare.

#### Need and/or Problem Identification

Fourteen (74%) of 19 frameworks emphasized the importance of identifying the needs and/or problems that the AI solution aims to address during the initial phases of technology development [1,51-63]. However, none of the frameworks provided any tools or guidelines to conduct this needs assessment. All international-level frameworks stated that AI in HS should enhance system capacity or improve health outcomes [1,51,56]. At the local level, 2 frameworks explicitly asserted that the use of AI should be based on a clearly defined need and an appropriate contextual understanding [54,62]. In a similar manner, 7 organizational-level frameworks stated that the need or problem an AI technology is intended to address should be clearly identified during the initial stage, prior to the development of the technology [52,55,57,58,60,61,63]. One of these frameworks also underscored the importance of understanding the context in which the problems arise, which should rely on the inclusion of frontline workers to describe the problems in an effective way [55]. In contrast, at the national level, only Parker et al [59] highlighted the importance of developers understanding the needs of HS to develop tailored AI-based technologies.

#### Data Governance

Although data security was addressed in 14 (74%) of 19 frameworks [1,30,52,54,56-60,62-66], only 11 (58%) frameworks included information on forms of consent [1,30,53,56-58,62-66], and merely 6 (32%) considered data quality [53-55,60,61,63]. For instance, all international-level frameworks emphasized the need for a well-established data protection structure, including clear laws and regulations frameworks to ensure transparency and individuals’ privacy [1,51,56]. The WHO’s frameworks underscore the importance of establishing clear procedures for obtaining informed consent from data subjects [1,56]. Similarly, all national-level frameworks refer to the development of procedures or frameworks to ensure data security [30,59,64-66]. Four of them mention concerns related to obtaining informed consent and patient privacy [30,64-66]. Nevertheless, none of the international or national frameworks reviewed address the issue of data quality. Local-level frameworks addressed at least two critical elements of data governance. Arnaout et al [62] and Whittaker et al [54] mentioned the need for ensuring appropriate and secure environments for data storage and sharing. Carter et al [53] and Arnaout et al [62] underscored the importance of explaining the use of data to patients and of ensuring that they understand and approve it. Additionally, Carter et al [53] and Whittaker et al [54] emphasized that data quality requires the use of data that are both complete and representative of the targeted population.

In the same vein, 5 organizational-level frameworks encompassed key data governance components related to data security [52,57,58,60,63]. Some of these components are privacy protection [57,58,63], secure storage mechanisms, management of datasets [60], and data traceability [58]. However, explicit recognition of the critical role of consent in protecting patients’ rights is found in only 2 organizational-level frameworks [57,63]. Four organizational-level frameworks highlight the importance of ensuring data quality [55,60,61,63], including the reduction of bias, as well as ensuring data diversity and appropriate data cleaning [55].

#### Risk Assessment and Management

Eighty-nine percent (17/19) of frameworks included risk assessment and management as part of AI governance and underscore the need for both proactive (predeployment) and reactive (postimplementation) strategies [1,30,50,52-64,66]. At the international level, the 2 WHO frameworks addressed privacy risks associated with the use of AI technologies in HS [1,56]. One of them highlighted that providers or developers should inform deployers about the potential risks of the AI technology, thereby allowing for an informed decision regarding its implementation [56]. Three national-level frameworks emphasized the importance of national standards and postmarket safety monitoring to ensure the timely identification and reporting of risks to patients [30,64,66], while for another 3 frameworks, risk assessment should be an ongoing process, supported by continuous monitoring of safety and risks [30,59,66]. Likewise, 2 local-level frameworks emphasized the need for assessing and managing risks prior to the deployment of AI-based technologies [54,62], and 2 frameworks pointed out the need for ongoing monitoring after deployment [53,62].

The same trend is observed in all 8 organizational-level frameworks [50,52,55,57,58,60,61,63]. Three of them bring forward the importance of implementing continuous monitoring of the algorithm [50,55,60]. Bedoya et al [61] outlined a risk-based triage system algorithm review, ranging from “full committee evaluation” to “no further review.” Economou-Zavlanos et al [63] emphasized an iterative approach that supports continuous feedback between reviewers and developers to strengthen risk-benefit assessments. Furthermore, Kim et al [55] advocated for organizations’ early identification and mitigation of potential AI-related risks.

#### Validation and/or Evaluation

Most frameworks highlighted validation and/or evaluation processes as core components of effective AI governance in HS [1,30,50-64,66]. The 3 international-level frameworks included these components [1,51,56]. While 2 of them highlighted the evaluation of AI performance as a crucial step prior to deployment [1,51], 1 emphasized the importance of validation in assessing the suitability of the data used in the models [56]. Equally, 4 national-level frameworks incorporated the validation and evaluation of AI-based technologies as essential steps prior to deployment and/or integration into HS [30,59,64,66]. For instance, Parker et al [59] noted that the evaluation process, which should be conducted by governance teams at the HS level but also by third parties providing assurance reviews, may encompass considerations such as performance, privacy, compliance, legal issues, patient safety, clinical integration, IT integration, and validation using internal datasets.

Three local-level [53,54,62] and 8 organizational-level [50,52,55,57,58,60,61,63] frameworks emphasized the importance of continuously evaluating AI-based technologies following implementation. Two local-level frameworks highlighted that validation and evaluation processes are essential for assessing the performance of AI-based technologies prior to their deployment in HS [53,62]. Three organizational-level frameworks emphasized that this evaluation should include the analysis of bias, potential integration into the clinical workflow, and data handling procedures [57,58,60]. In addition, 3 organizational-level frameworks underscored that an initial evaluation should be conducted to assess regulatory approval needs, strategic alignment with the organization, and resource requirements, including funding, time, data environment, infrastructure, and human capital [55,58,60].

In the same manner, all organizational-level frameworks included validation or evaluation of AI-based technologies in the development and/or deployment stages. These frameworks also shared a consensus on the importance of validating datasets to determine the appropriateness of the AI technology for the target population [50,52,55,57,58,60,61,63]. However, not all organizational-level frameworks specified the strategies for doing so [50,57,58,61,63]. For instance, 3 of them supported the use of “real-world clinical data” for evaluation, recommending an approach in which the AI technology is used, but its results remained hidden from clinical users during the assessment phase [55,61,63]. Finally, 3 organizational-level frameworks outlined a set of metrics for postdeployment evaluation, such as algorithm sensitivity and specificity, patient outcomes, reduction in burden on HS providers, and overall cost efficiency [50,55,60].

#### Maintenance and Monitoring

Eighty-four percent (16/19) of the frameworks emphasized maintenance and monitoring in AI governance for HS [1,30,50-55,57,59-64,66]. At the international level, the WHO’s framework underlines that robust postmarketing surveillance could enhance regulatory oversight by helping to identify biases [1]. Similarly, Morley et al [51] highlighted that collective intelligence at this level can be supported through national oversight procedures, as establishing such knowledge sharing mechanisms would enable countries to access information about AI-based technologies they are considering or have already implemented. However, the manner to integrate monitoring varies among national-level frameworks [30,59,64,66]. While 2 frameworks suggested integrating regular audits and reporting to enable ongoing safety monitoring [30,59], 2 others emphasized the implementation of a national postmarket safety monitoring system [64,66]. Furthermore, local-level frameworks emphasized the need to establish reporting mechanisms following the implementation of AI-based technologies [53,54], as well as continuous monitoring for biases, errors, and performance assessment [53,62].

Correspondingly, 7 organizational-level frameworks highlighted maintenance and monitoring in AI governance [50,52,55,57,60,61,63]. Four frameworks emphasized that performance and impact should be monitored regularly to detect performance drift and assess clinical outcomes after implementation [50,55,61,63]. Likewise, 2 organizational-level frameworks underlined the crucial role of monitoring outcomes related to safety, as well as clinical and technical performance [61,63]. Nevertheless, only 2 frameworks pointed out that monitoring should also include equity in care, economic impact, and workforce satisfaction [55,60].

#### Integration

Although 47% (9/19) of the frameworks emphasized integrating AI-based technologies into HS as part of governance processes at national [30,66], local [54], and organizational levels [52,55,58,60,61,63], none of the international frameworks mentioned this integration. In all these frameworks, “integration” refered to the analysis of how the new AI technology will align with or affect existing clinical workflows [30,52,54,55,58,60,61,63,66]. The national-level frameworks recommend establishing a clinical governance committee to support the integration of AI-based technologies into clinical workflows [30,66]. At the local level, Whittaker et al [54] pointed out that involving clinicians in the conception and development phases helps to better understand how seamlessly new technologies can integrate into clinical workflows. According to them, without insight into how local health services currently operate, new developments may entirely fail to meet the practical needs of those systems. Likewise, organizational-level frameworks emphasized the importance of understanding current workflows [63], designing intuitive user interfaces, supporting frontline workers [55], and adapting AI-based technologies during deployment to ensure its seamless integration and optimal use [52,60].

### Governance Relational Mechanisms

#### Overview

Relational mechanisms are process elements that foster trust, participation, partnership, and mutual understanding. Within the included frameworks, 4 relational mechanisms were identified as critical: ethical principles and/or values, education and training, communication, and standards and regulations. Table 3 summarizes the percentage of frameworks that included these relational mechanisms.

**Table 3.** Artificial intelligence governance relational mechanisms identified across the included frameworks.

Author and year	Ethical principles and/or values^a^	Education and training^b^	Communication^c^	Standards and regulations^d^
International level				
WHO^e^, 2024 [56]	X	X	X	X
Morley et al, 2022 [51]		X		X
WHO, 2021 [1]	X	X	X	X
National level				
Reddy et al, 2020 [30]	X	X		X
Jaremko et al, 2019 [65]		X		
Parker et al, 2024 [59]	X	X	X	
AAAiH^f^, 2023 [64]	X	X	X	X
Solaiman, 2025 [66]	X		X	X
Local level				
Arnaout et al, 2024 [62]	X	X	X	
Whittaker et al, 2023 [54]	X	X		X
Carter et al, 2024 [53]	X	X	X	
Organizational level				
Liao et al, 2022 [50]	X			X
Bedoya et al, 2022 [61]	X	X	X	X
Hassan et al, 2025 [58]	X	X		X
Economou-Zavlanos et al, 2024 [63]	X		X	X
Daye et al, 2022 [57]	X		X	X
Kim et al, 2023 [55]	X		X	
Apfelbacher et al, 2024 [52]	X	X		
Kim et al, 2026 [60]	X	X	X	X

a Total frameworks: 89%.

b Total frameworks: 74%.

c Total frameworks: 63%.

d Total frameworks: 68%.

e WHO: World Health Organization.

f AAAiH: Australian Alliance for Artificial Intelligence in Healthcare.

#### Ethical Principles and/or Values

Although 89% (17/19) of the frameworks integrated principles and/or values into AI governance for HS [1,30,50,52-64,66], most commonly emphasizing the importance of ethical principles, such as privacy, inclusiveness, fairness, and transparency [1,30,50,52,54,56-60,62-64,66], they reflected a heterogeneous understanding of how these principles are embedded within AI governance. At the international level, the 2 WHO frameworks placed ethics and human rights at the core of AI governance and highlighted that achieving this goal requires both government interventions and actions from companies [1,56]. Therefore, they recommend companies to adopt a paradigm that grounds technology design in “the values of human dignity, freedom, equality, and solidarity” [1,56]. Additionally, these frameworks also emphasized the importance of inclusiveness as a core principle for achieving ethics-centered AI governance. This includes integrating community oversight [1,56] and ensuring the participation of low- and middle-income countries [56], as well as indigenous communities [1], in shaping international AI governance standards. Finally, 1 framework also highlighted human autonomy and responsiveness principles [1].

At the national level, 3 frameworks suggested different approaches to integrate ethics into AI governance [30,59,64]. While the Australian Alliance for Artificial Intelligence in Healthcare (AAAiH) advocated for the establishment of a national ethical framework [64], Parker et al [59] emphasized that a comprehensive AI governance, guided by an interdisciplinary committee including expertise in ethics, can foster adherence to ethical standards. Likewise, Reddy et al [30] argued that a governance model based on the principles of fairness, transparency, trustworthiness, and accountability can address ethical challenges such as biases, privacy concerns, and trust of both the clinicians and the public in the use of AI in HS.

At the local level, 2 frameworks emphasized the importance of using training datasets that are representative and inclusive of population diversity. They also highlighted the need for AI governance that considers societal impacts, extending beyond individual risks and benefits [53,54]. While one of them emphasized that ethics should be embedded throughout the design and development of AI-based technologies [54], the other underscored the need for ethical review processes to assess how clinicians and patients are likely to engage with model outputs in clinical decision-making [53]. Additionally, Whittaker et al [54] asserted that AI governance should demonstrate respect for traditional knowledge and cultural protocols.

At the organizational level, all frameworks incorporated ethical principles in AI governance [50,52,55,57,58,60,61,63]. Three of these frameworks explicitly emphasized the importance of integrating ethical principles before and/or during the deployment of AI-based technologies [50,57,58]. Therefore, AI governance bodies within the organization are accountable for ensuring that both internal or external developers integrate these principles. For instance, Daye et al [57] argued that during the preimplementation assessment process, the committee must consider fairness and other ethical issues and address systemic biases, including those affecting disadvantaged communities. Similarly, Economou-Zavlanos et al [63] incorporated ethical and quality principles across the development lifecycle by introducing “evaluation checkpoints” where principles of clinical value, safety, fairness, and equity are systematically reevaluated throughout the development process. Regarding equity, 4 frameworks explicitly identified it as an outcome to be measured in AI governance [50,55,57,60]. Liao et al [50] and Kim et al [55] emphasized the importance of ensuring an equitable distribution of the benefits across population subgroups [50,55]. Furthermore, 2 frameworks highlighted the need for equitable resource allocation for AI implementation to avoid exacerbating existing disparities between units or departments [55,57].

#### Education and Training

Seventy-four percent (14/19) of the frameworks incorporated education and training as key mechanisms of AI governance. All these frameworks emphasized the need to educate and train professionals in the use of AI-based technologies within HS [1,30,51-54,56,58-62,64,65]. Two international-level [1,56] and 3 national-level [30,64,65] frameworks also highlighted the importance of improving AI literacy among the general public, including patients. Moreover, 1 international-level framework highlighted that providing education and training in ethics to designers and developers is critical to AI governance [1]. At the local level, Whittaker et al [54] advocated for technical guidance that reflects the specific needs and conditions of the local context. In addition, 2 organizational-level frameworks underscored the importance of implementing training plans, including the availability of materials to educate end users on the appropriate use of AI-based technologies [61,63].

#### Communication

Communication is identified as a key mechanism in 63% (12/19) of the frameworks [1,53,55-57,59-64,66]. Although communication concerns vary across all levels of governance, 5 frameworks recognize the role of effective communication in improving transparency about the use of AI in HS [53,56,59,62,64]. For instance, 1 national-level framework suggested that HS must ensure clear communication strategies to inform when AI-based technologies are being considered, so they can bring them into the governance process [59]. Likewise, 1 local-level framework stressed that AI users should receive clear, transparent, and context-specific information to reduce the potential risk of misuse and foster trust in the AI technology [62]. At the organizational level, 5 frameworks highlighted the need to establish clear and consistent communication between developers and the governance committee [55,57,60,61,63]. Two of them included the end users in this dynamic of communication to improve the AI technology [55,57].

#### Standards and Regulations

Sixty-eight percent (13/19) of the frameworks incorporate standards and regulations in AI governance for HS [1,30,50,51,54,56-58,60,61,63,64,66]. Two international-level frameworks emphasized the importance of establishing international standards to reduce risks to patient safety [1,51]. Likewise, 3 international-level and 2 national-level frameworks brought forward the need to create national standards to support the development and deployment of AI [1,51,56,64,66], ensuring safety, quality, and ethical standards. For instance, Reddy et al [30] argued that normative standards include “how AI models will be designed and deployed” in the context of HS. At the organizational level, of the 6 frameworks that address standards and regulations concerns [50,57,58,60,61,63], 3 proposed a “risk-based” approach to evaluation of AI models, with regulatory requirements that vary according to technology type and potential risks [57,61,63].

### Governance Barriers and Challenges

Fifty-three percent (10/19) of the frameworks identified barriers and challenges to AI governance for HS [1,50,51,54,55,57,59,60,63,66]. International-level frameworks highlighted challenges arising from the disconnection between different yet highly interrelated policy domains across governance levels, the lack of international coordination in AI governance for HS [51], and concerns about the potential emergence of a “market” in which individuals can “buy and sell health data” [1]. Across other levels, 6 frameworks emphasized challenges related to the resources (eg, costs, infrastructure, and time) required for the effective implementation of AI governance [54,57,59,60,63,66]. Additionally, 3 organizational-level frameworks raised concerns about the ongoing resource demands necessary for the continuous monitoring of AI-based technologies [55,57,60].

### An Integrative AI Governance Model for HS

**Figure 4.**
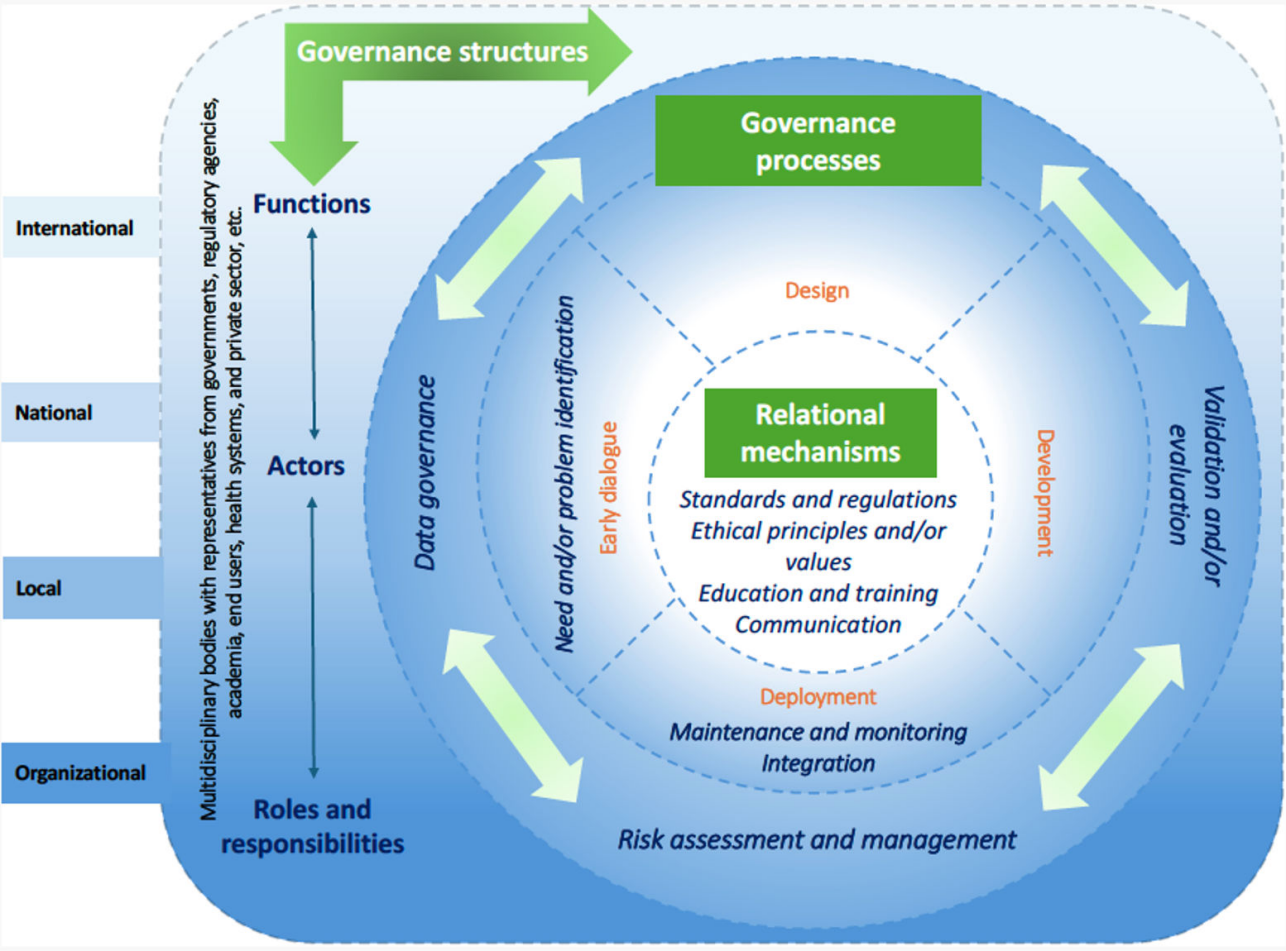
The Integrative AI Governance Model for HS. Lighter colors show more heterogeneity in the findings, while darker colors emphasize more consistency in the literature.

None of the reviewed frameworks provided a comprehensive approach that fully captures the multidimensional and dynamic nature of AI governance in HS. We therefore synthesized the dimensions and components reported across the 19 included frameworks to propose an integrative AI governance model that identifies key elements to guide AI-related policy, practice, and research in HS. The resulting model is presented in Figure 4. The model advances the field by explicitly articulating AI governance as a multilevel, interrelated system, in contrast to existing health information system or responsible AI governance frameworks that typically address only selected components or specific settings. Importantly, this integrative model reflects the current state of the literature and is not intended as a ready-to-implement governance framework for decision-makers. Rather, it provides a foundational scaffold that requires further refinement through expert validation, stakeholder and end user consultation, pilot testing, and evaluation.

The integrative AI governance model for HS shows how the key governance components identified across the included frameworks are deeply interconnected, as well as the permeability of different levels of AI governance. The framework comprised 2 governance dimensions (structures and processes) and 4 relational mechanisms (ethical principles and/or values, education and training, communication, and standards and regulations) in 4 interconnected levels (organizational, local, national, and international). The darker blue at the organizational level reflects a higher concentration of well-established and more specific AI governance frameworks for HS. For instance, the AI governance functions vary across levels but follow a complementary pattern.

The double-headed arrows highlight the dynamic nature of interactions between the dimensions and their components. For instance, the bidirectional arrow linking the structural elements (functions, actors, and roles and responsibilities) illustrates a reciprocal relationship. AI governance functions are shaped by actors who hold distinct yet complementary (and sometimes conflicting) roles and responsibilities within multidisciplinary bodies. These actors play a decisive role in defining, operationalizing, and adapting governance functions. Conversely, the governance structure and its functions can also shape, redefine, or shift the roles, relative importance, and influence of these actors over time. Similarly, the 2-way arrows between data governance, risk assessment and management, and validation and/or evaluation illustrate the interdependence among these components across the entire AI lifecycle. These 3 components are intended to ensure that AI-based technologies fulfill their goals while safeguarding public health, and individual privacy and safety.

The gradient from blue to white at the center of the processes represents the adaptation and the interaction between the relational mechanisms arising from the interplay among all components of the AI governance framework for HS. Thus, establishing ethical principles, standards, and regulations, together with clear, context-sensitive, and transparent communication strategies, as well as tailored education and training programs and initiatives adapted to different audiences (eg, health professionals, policy-makers, managers, patients, and the general population), is essential at international, national, local, and organizational levels to guide the responsible development and deployment of AI-based technologies within HS [1,30,56,64], particularly given their potential to reflect and reproduce structural inequities [67,68]. Nevertheless, their adoption and adaptation over time can differ depending on the context characteristics and resources, potentially giving rise to new relational mechanisms within AI governance processes. The 4 relational mechanisms underpin all processes, especially need and/or problem identification, maintenance and monitoring, and integration, which are critical to ensure AI and HS sustainability.

## Discussion

### Principal Findings

This study synthesized existing AI governance frameworks for HS published over the last decade and reviewed 19 frameworks distributed across international, national, local, and organizational levels. The frameworks articulated operational arrangements meant to translate ethical principles and legal and regulatory requirements into actionable structures, processes, and mechanisms. Consistent with prior literature reviews [10,69,70], our findings highlighted that despite the growing number of AI governance frameworks for HS, quality and implementability are largely lacking.

### Contribution to the Literature

#### Integrative AI Governance Model for HS

This study contributed an integrative AI governance model for HS, which gathers 6 processes and 4 relational mechanisms across the 4 governance levels, explicitly modeling their interactions. Although no previous integrative framework has been proposed against which comparisons can be made, prior published general AI governance frameworks underscored similar structures, processes, and mechanisms [10,35,71]. For instance, the responsible AI governance framework of Papagiannidis et al [10] includes dimensions of privacy, data governance, safety, and bias, which are aligned with our integrative framework. Furthermore, the governance framework of Stogiannos et al [37] emphasized that HS staff training should include core AI concepts, including basic principles, model validation and evaluation, clinical applications, ethical considerations, regulatory requirements, and AI model limitations. Although this framework shared certain features with ours, it is primarily designed to inform procurement decisions [37].

#### Theoretical Grounding Between AI Governance and HS Governance

By proposing a multidimensional and multilevel framework, our integrative model provides a foundation to better integrate an interdisciplinary analytical lens to AI governance in HS, namely, governance theories and models anchored in the social sciences that can inform current debates on the most appropriate governance arrangements for HS [72-74].

First, robust governance is widely recognized as a central determinant of HS performance and resilience [72,75-77]. A large body of literature suggests that, among settings with comparable resources, differences in HS performance are often explained less by financing, infrastructure, or workforce levels than by the quality of governance mechanisms [72,75-77]. Conversely, dysfunctions, such as corruption, poor coordination, weak accountability mechanisms, and competing agendas, can severely undermine HS effectiveness, even when substantial financial and technical investments are made [72,75-77]. The analysis of AI governance in HS must therefore pay closer attention to the multilevel dynamics that structure these systems, particularly the institutional logics shaping governance arrangements and practices within the AI ecosystem [78-80].

Second, a multilevel analytical perspective is also warranted as HS are characterized by institutional pluralism, bringing together diverse stakeholders, such as governments, regulatory bodies, technology providers, investors, scientists, health professionals, patients, and civil society [74]. While interactions among these actors are shaped by power relations and trade-offs, several studies indicate that current AI governance in HS remains largely technocratic, overlooking the sociopolitical, economic, and even ideological dynamics inherent in AI development and use [1,81]. In this context, governance transcends the mere regulation of a technological tool; it requires arbitrating among competing (sometimes antagonistic) objectives and priorities to protect the public interest [73,82-85]. Finally, HS research offers conceptual frameworks that support a more structured analysis of interactions among stakeholders across multiple levels of governance [72,75-77]. These approaches also help examine how institutions shape relationships among actors and how resources are allocated, accessed, and mobilized within the governance system [72,75-77].

### Further Research and Key Improvement Opportunities

While the proposed integrative model constitutes an important synthesis of knowledge about the governance of AI in HS, there remains significant scope for improvements.

#### Improving Quality

To improve quality, further research should use systematic and transparent methods, such as case studies, network analysis, and stakeholder mapping, to guide framework development and implementation and to analyze specific actors’ roles and responsibilities [86-90]. In addition, data governance should be expanded beyond data security to encompass patient consent and data quality, as even the most advanced AI-based technologies depend on high-quality datasets to function effectively [91]. Data security overshadowing data quality in current governance frameworks is a key insight of this study, as dataset quality can have a significant impact on mitigating bias and ensuring equity in the delivery of care using AI technologies [92-94]. Data security should also be broadened to proactively address cyberattacks, particularly ransomware targeting health data, as well as issues of data sovereignty [1,7,25,26,56]. Given that such threats and issues are expected to increase, integrating them into AI governance within HS is essential [95-97]. Finally, to improve comparability and rigor, further research should make AI validation and evaluation and maintenance and monitoring metrics available across all AI model levels, and include equity, economic, and environmental indicators [98-100].

#### Improving Processes

Further research is needed to better understand the governance process of “integration” at a broader level as AI requires a systemic transformation, which goes beyond individualized concerns for workflow redesign or workforce adaptation [7,101]. While these elements are important, they should be situated within a broader (architectural) reorganization of HS [13,25]. For example, a review examining the application of AI in radiology showed that, despite AI’s substantial potential to optimize patient care, its effective use requires a reorganization of professional roles and responsibilities, as well as a redesign of regulatory frameworks [102]. Specifically, clinicians need to learn to develop competencies to supervise and appropriately use AI systems, while regulatory frameworks must evolve to balance requirements for human oversight with the increasing autonomy and agency of AI models [102]. The current emphasis on adaptation rather than systemic transformation appears misaligned with the broader ambitions often attributed to AI-based technologies [101]. This underscores the need for future studies to analyze and inform the systemic transformation within HS to fully benefit from AI.

#### Improving Mechanisms

Regarding the relational mechanisms, 4 main areas for improvement were identified. First, there is a need to better conceptualize and operationalize the ethical principles and/or values specific to AI in HS [103]. As many AI-based technologies reproduce and reinforce health inequities [104], scholars have underscored the need for tools that help stakeholders integrate ethical principles and/or values in emerging technologies [103,105]. Future governance frameworks could build a more constructive dialogue with previous reviews about AI ethical principles and/or values to strengthen their conceptualization and operationalization [16,17,103,106]. For instance, according to Fields et al [106], addressing racial bias in AI-based technologies requires concrete measures, including the meaningful inclusion of Black researchers, other minority scholars, and community members throughout the AI development process. It also entails conducting audits explicitly designed to detect and mitigate racial (and other) bias, strengthening transparency, and establishing robust accountability mechanisms [106].

Second, there is a need to clarify whether standards and regulations should be legally binding or merely aspirational [7]. On this issue, the WHO calls for the urgent development of governance frameworks for AI that safeguard the rights of individuals and communities, promote equity and justice, while guiding innovation and strengthening the resilience of HS [1]. Such governance must be grounded in local sociopolitical, institutional, and cultural realities, supported by robust legal, regulatory, and accountability mechanisms, and responsive to the operational challenges associated with integrating AI into health care practice [20-22].

Third, AI education and training programs within HS governance will require adaptable strategies that can keep pace with rapid developments in AI, ensuring that professionals are equipped with up-to-date knowledge and skills. These initiatives should also equip policymakers, managers, patients, the general public, and other stakeholders directly or indirectly affected by the use of AI in health [1,7,14,56]. Fourth, further research should clarify the actors engaged in communication, the direction and flow of that communication, and the specific target audiences (eg, clinicians, patients, and public), as this dimension remains insufficiently analyzed and developed [107,108]. In sum, to strengthen the model’s institutional and practical relevance, future research should prioritize the validation and operationalization of the integrative AI governance model. This agenda could be advanced through Delphi studies involving diverse stakeholders across multiple HS levels, complemented by case studies conducted in varied geographic, sociopolitical, cultural, and economic contexts to refine the model and support its broader applicability [90,109-111].

### Strengths and Limitations of the Study

To the best of our knowledge, this systematic review is the first to synthesize existing AI governance frameworks across 4 governance levels while simultaneously assessing their quality. Yet, this study has certain limitations. First, although our search strategy was comprehensive and extended to gray literature, it remains possible that frameworks in progress or those disseminated in other languages were not identified. Regarding language restrictions, we acknowledged that relevant frameworks may exist in other languages, particularly in countries that are advanced in AI development (eg, China and Germany). However, we did not conduct literature searches in these languages as our team does not possess the linguistic expertise required to analyze documents in these languages. Relying on automated translation tools was not considered an appropriate option, as these tools pose a substantial risk of misinterpreting concepts and terms whose meaning depends heavily on context. Consequently, our model carries a Western bias, as the underlying evidence is primarily sourced from the Global North. This disparity underscores a critical need for future research to investigate AI governance within contexts of the Global South.

Second, a limitation arises from the use of an adapted version of AGREE-HS to evaluate the methodological quality of each included framework. Although this tool is the best option to evaluate whether the framework’s authors explicitly define the topic (what), participants (who), and methods (how), several AGREE-HS items are interpretative in nature requiring contextual judgment. However, we ensured the robustness and consistency of data extraction and appraisal through independent coding, calibration, and consensus-based adjudication. This approach is commonly adopted in reviews incorporating qualitative appraisal, where calibration, discussion, and consensus are recognized as appropriate strategies to ensure methodological rigor [112]. Finally, the heterogeneity of this body of literature may have constrained the extent to which the distinctive nuances of each framework were fully captured in the results. To mitigate this limitation, our review and our model were developed systematically, following rigorous methodological guidance [39], to better examine diverse frameworks spanning multiple AI governance components and contexts.

### Conclusions

This systematic review of AI governance frameworks for HS highlights key elements of AI governance structures, processes, and mechanisms distributed across international, national, local, and organizational levels. Rather than focusing on specific governance dimensions or settings, the review innovates by integrating all governance components and their interactions across contexts. This study thus differs from other reviews by contributing a multilevel analysis that elucidates the strengths and gaps within current frameworks, adding to this expanding body of scholarship. The study brings to the field an integrative AI governance model for HS. The proposed model may inform future scholarly work as well as real-world policy and practice-oriented discussions on ensuring the responsible and sustainable integration of AI-based technologies into HS.

## Supplementary material

10.2196/87448 Multimedia Appendix 1 Literature search strategy.

10.2196/87448 Multimedia Appendix 2 Modifications to the Appraisal of Guidelines for Research and Evaluation for HS (AGREE-HS).

10.2196/87448 Multimedia Appendix 3 Study characteristics (n=19).

10.2196/87448 Multimedia Appendix 4 Characteristics of the AI governance frameworks for HS.

10.2196/87448 Multimedia Appendix 5 AI governance structure functions identified across the included frameworks.

10.2196/87448 Multimedia Appendix 6 Actors’ roles and responsibilities.

10.2196/87448 Checklist 1 PRISMA checklist.

## Abbreviations

AAAiH: Australian Alliance for Artificial Intelligence in Healthcare
AGREE-HS: Appraisal of Guidelines for Research and Evaluation for Health Systems
AI: artificial intelligence
HS: health systems
PRISMA: Preferred Reporting Items for Systematic Reviews and Meta-Analyses
SSRN: Social Science Research Network
WHO: World Health Organization
