# Supplementary material for: Artificial Intelligence Governance in Health Systems: Systematic Review of Frameworks and Integrative Model Proposal
Source: J Med Internet Res. 2026 Jun 8;28:e87448. doi: 10.2196/87448 (PMC13245845; doi:10.2196/87448)
Supplement: Multimedia Appendix 1 [file jmir-v28-e87448-s001.pdf]

Appendix 1. Literature Search Strategy

Academic literature

Concept 1: Artificial intelligence
Concept 2: Governance
Concept 3: Health system

Filter 1: in the last 10 years
Filter 2: English, French, Spanish, Portuguese
Filter 3: Humans

Database	Search strategy
**PubMed**	(("Artificial Intelligence"[MeSH:NoExp] OR "Machine Learning"[MeSH:NoExp] OR "Natural Language Processing"[MeSH:NoExp]) OR ("Artificial Intelligence"[Title/Abstract] OR "AI"[Title/Abstract] OR "machine learning"[Title/Abstract] OR "deep learning"[Title/Abstract] OR "Natural Language Processing"[Title/Abstract]) OR ("Artificial Intelligence" OR "AI" OR "machine learning" OR "deep learning"))<br>**AND**<br>(("Health Policy"[MeSH:Exp] OR "Public Policy"[MeSH:Exp] OR "Health Services Administration"[MeSH:Exp] OR "Ethics"[MeSH:Exp] OR "Morals"[MeSH:Exp] “Guidelines as Topic**”** [MeSH:NoExp]) OR ("AI Governance"[Title/Abstract:~3] OR "AI Regulation"[Title/Abstract:~3] OR "AI Framework"[Title/Abstract:~3] OR "AI Model"[Title/Abstract:~3] OR "Artificial Intelligence Guideline"[Title/Abstract:~3] OR "Ethic"[Title/Abstract] OR "AI principles"[Title/Abstract:~3] OR "responsible innovation"[Title/Abstract] OR "Governance Framework"[Title/Abstract:~3]) OR ("AI Governance" OR "AI Framework" OR "Governance Model" OR "Guideline" OR "Ethic" OR "AI principles" OR "AI regulation" OR "Governance Framework"))<br>**AND**<br>**(**("Health Care Quality, Access, and Evaluation"[MeSH:Exp] OR "Delivery of Health Care"[MeSH:Exp] OR "Public Health Infrastructure"[MeSH:Exp]) OR "Healthcare System"[Title/Abstract] OR "Health Services"[Title/Abstract] OR "Health Sector"[Title/Abstract] OR "Hospital"[Title/Abstract] OR "Healthcare Delivery"[Title/Abstract] OR "Public Health Systems"[Title/Abstract]))

**MEDLINE (Ovid)**	("Artificial Intelligence"/ OR "Machine Learning"/ OR "Natural Language Processing"/) OR ("Artificial Intelligence".ti,ab. OR "AI".ti,ab. OR "machine learning".ti,ab. OR "deep learning".ti,ab. OR "Natural Language Processing".ti,ab.) OR ("Artificial Intelligence" OR "AI" OR "machine learning" OR "deep learning")<br>**AND**<br>("Health Policy"/ OR "Public Policy"/ OR "Health Services Administration"/ OR "Ethics"/ OR "Morals"/ OR "Guidelines as Topic"/) OR (AI adj3 (Govern* OR Regulat* OR Framework* OR Model* OR Guide* OR Ethic* OR princip* OR respons*)) OR ("AI Governance" OR "AI Framework" OR "Governance Model" OR "Guideline" OR "Ethic" OR "AI principles" OR "AI regulation" OR "Governance Framework")<br>**AND**<br>("Health Care Quality, Access, and Evaluation"/ OR "Delivery of Health Care"/ OR "Public Health Infrastructure"/) OR ("Health System".ti,ab. OR "Health Services".ti,ab. OR "Health Sector".ti,ab. OR "Hospital".ti,ab. OR "Healthcare Delivery".ti,ab. OR "Public Health Systems".ti,ab.)
**Embase (Ovid)**	"Artificial Intelligence"/ OR "Machine Learning"/ OR "Natural Language Processing"/ OR ("Artificial Intelligence" OR "AI" OR "machine learning" OR "deep learning" OR "Natural Language Processing").ti,ab,kw<br>**AND**<br>**(**("Health Care Policy"/ OR "Public Policy"/ OR "Health Service"/ OR "Ethics"/ OR "Morals"/ OR "Guidelines as Topic"/) OR (AI adj3 (Govern* OR Regulat* OR Framework* OR Model* OR Guide* OR Ethic* OR princip* OR respons*)).ti,ab,kw OR ("AI Governance" OR "AI Framework" OR "Governance Model" OR "Guideline" OR "Ethic" OR "AI principles" OR "AI regulation" OR "Governance Framework").ti,ab,kw<br>**AND**<br>("Health Care"/ OR "Delivery of Health Care"/ OR "Health Infrastructure"/) OR (Health adj2 (System* OR Sector* OR Hospital* OR Delivery*)).ti,ab,kw
**ACM Digital Library**	Abstract: ("artificial intelligence" OR "ai" OR "machine learning" OR "deep learning" OR "natural language processing")<br>**AND**

	Abstract: ("governance" OR "regulation" OR "framework" OR "model" OR "policy" OR "guideline" OR "ethic" OR "standard" OR "principles" or "regulatory" OR "responsible")<br>**AND**<br>Abstract: ("health system" OR "healthcare system" OR "health services" OR "health sector" OR "hospital" OR "healthcare delivery" OR "public health systems") “filter”: E-Publication Date: (11/01/2014 TO 10/31/2024), ACM Content:DL}
**Web of Science (Clarivate)**	TS=(("Artificial Intelligence" OR "Machine Learning" OR "Natural Language Processing" OR "AI" OR "deep learning"))<br>**AND**<br>TS=(("Health Policy" OR "Public Policy" OR "Health Services Administration" OR "Ethics" OR "Morals" OR "Guidelines as Topic"/) OR (AI NEAR/3 (Govern* OR Regulat* OR Framework* OR Model* OR Guide* OR Ethic* OR princip* OR respons*)) OR ("AI Governance" OR "AI Framework" OR "Governance Model" OR "Guideline" OR "Ethic" OR "AI principles" OR "AI regulation" OR "Governance Framework"))<br>**AND**<br>TS=(("Health Care" OR "Delivery of Health Care" OR "Public Health Infrastructure") OR ("Health System" OR "Health Services" OR "Health Sector" OR "Hospital" OR "Healthcare Delivery" OR "Public Health Systems"))
**Scopus**	TITLE-ABS-KEY("Artificial Intelligence" OR "AI" OR "machine learning" OR "deep learning" OR "Natural Language Processing")<br>**AND**<br>TITLE-ABS-KEY (("Health Policy" OR "Public Policy" OR "Health Services Administration" OR "Ethics" OR "Morals" OR "Guidelines as Topic"/) OR (AI W/3 (Govern* OR Regulat* OR Framework* OR Model* OR Guide* OR Ethic* OR princip* OR respons*)) OR ("AI Governance" OR "AI Framework" OR "Governance Model" OR "Guideline" OR "Ethic" OR "AI principles" OR "AI regulation" OR "Governance Framework"))<br>**AND**<br>TITLE-ABS-KEY ("Health System" OR "Healthcare System" OR "Health Services" OR "Health Sector" OR "Hospital" OR "Healthcare Delivery" OR "Public Health Systems")

**PsycINFO (EBSCO)**	("Artificial Intelligence"/ OR "Machine Learning"/ OR "Natural Language Processing"/)<br>OR ("Artificial Intelligence".ti,ab. OR "AI".ti,ab. OR "machine learning".ti,ab. OR "deep learning".ti,ab. OR "Natural Language Processing".ti,ab.) OR ("Artificial Intelligence" OR "AI" OR "machine learning" OR "deep learning")<br>**AND**<br>("Health Policy"/ OR "Public Policy"/ OR "Health Services Administration"/ OR "Ethics"/ OR "Morals"/ OR "Guidelines as Topic"/) OR (AI adj3 (Govern* OR Regulat* OR Framework* OR Model* OR Guide* OR Ethic* OR princip* OR respons*)) OR ("AI Governance" OR "AI Framework" OR "Governance Model" OR "Guideline" OR "Ethic" OR "AI principles" OR "AI regulation" OR "Governance Framework")<br>**AND**<br>("Health Care Quality, Access, and Evaluation"/ OR "Delivery of Health Care"/ OR "Public Health Infrastructure"/) OR ("Health System".ti,ab. OR "Health Services".ti,ab. OR "Health Sector".ti,ab. OR "Hospital".ti,ab. OR "Healthcare Delivery".ti,ab. OR "Public Health Systems".ti,ab.)
**Social Sciences Abstracts (EBSCO)**	"Artificial Intelligence"/ OR "Machine Learning"/ OR "Natural Language Processing"/ OR ("Artificial Intelligence" OR "AI" OR "machine learning" OR "deep learning" OR "Natural Language Processing")<br>**AND**<br>("Health Care Policy"/ OR "Public Policy"/ OR "Health Service"/ OR "Ethics"/ OR "Morals"/) OR (AI adj2 (Govern* OR Regulat* OR Framework* OR Model* OR Guide* OR Ethic* OR princip* OR respons*)).ti,ab. OR ("AI Governance" OR "AI Framework" OR "Governance Model" OR "Guideline" OR "Ethic" OR "AI principles" OR "AI regulation" OR "Governance Framework")<br>**AND**<br>("Health Care"/ OR "Delivery of Health Care"/ OR "Health Infrastructure"/) OR (Health adj2 (System* OR Sector* OR Hospital* OR Delivery*))

Grey Literature

Website	Link
Google Scholar	https://scholar.google.com
ProQuest Dissertations and Theses Database	https://www.proquest.com/dissertations/fromDatabasesLayer
Social Science Research Network	https://www.ssrn.com/ssrn/
Evidence for Informed Health Policymaking	https://www.pdq-evidence.org
World Health Organization	https://www.who.int
United Nations	https://www.un.org/fr/
Organisation for Economic Co-operation and Development	https://oecd.ai/en/
World Bank	https://www.worldbank.org/ext/en/home
European Union	https://european-union.europa.eu/index_en
National Institute for Health and Care Excellence	https://www.nice.org.uk
National Institute for Health	https://www.nih.gov

This is a Multimedia Appendix to a full manuscript published in the J Med Internet Res. For full copyright and citation information see https://www.jmir.org/2026/1/e87448

Alami H, Pozelli Sabio R, Pérez EJ, Gagnon MP, Langlois L, Denis JL, Malas K, Rivard L, Salvodelli M, Ag Ahmed MA, Fortin JP
Artificial Intelligence Governance in Health Systems: Systematic Review of Frameworks and Integrative Model Proposal
J Med Internet Res 2026;28:e87448
