# Supplementary material for: Artificial Intelligence Governance in Health Systems: Systematic Review of Frameworks and Integrative Model Proposal
Source: J Med Internet Res. 2026 Jun 8;28:e87448. doi: 10.2196/87448 (PMC13245845; doi:10.2196/87448)
Supplement: Multimedia Appendix 2 [file jmir-v28-e87448-s002.pdf]

Appendix 2. Modifications to the Appraisal of Guidelines for Research and Evaluation for HS (AGREE-HS)

This table summarizes the modifications made to the tool. Original criteria in red were removed or merged with other criteria, and the predefined scores for each criterion are shown in green.

Item	Original criteria for guidelines research and evaluation	Adapted tool for this project
**Topic:** This item addresses the description of the AI governance challenge, the causes of the challenge and the priority accorded to it, and the relevance of the guidance.	a. The health system challenge is clearly described (i.e., the nature of the challenge; the magnitude, frequency or intensity of the challenge; the populations affected).<br>b. The causes of the health system challenge are clearly described.<br>c. The health system challenge is described in terms of its level of priority in the targeted health system and the affected population; arguments to support the priority classification are provided.<br>d. The guidance is relevant to (i.e., timely in relation to when decisions will be made) and appropriate for: the health system challenge, the system or sub-system needs, the target population(s), and the setting(s) in which they will operate.	a. The AI governance challenges in the health system are clearly described. **(if yes = 2 points)**<br>b. The causes of the AI governance challenges in the health system are clearly described. **(if yes = 2 points)**<br>c. The framework/guidance is appropriate for the target population(s), and the setting(s) in which they will operate. **(if yes = 3 points)**
**Participants:** This item addresses the composition of the guidance development team and the management of competing interests and funder influence.	a. The health system guidance development team includes members who have an interest or stake in the recommendations (e.g., decision makers, program managers, operational leaders, consumers and members of the public).<br>b. The health system guidance development team is multidisciplinary (e.g., political scientists, economists, epidemiologists, methodologists).<br>c. The health system guidance development team is multi-sectoral (e.g., primary care, public health and, if appropriate to the challenge, finance and housing).<br>d. Competing interests of the health system guidance development team members (e.g., financial, professional) and the strategies used to identify and manage them, are clearly described.<br>e. Precautions have been taken to avoid or to minimize the influence of a funding agency.	a. The framework/guidance development team includes members who have an interest or stake in the recommendations. **(if yes = 2 points)**<br>b. It is multidisciplinary and or multi-sectoral. **(if yes = 2 points)**<br>d. Competing interests of the framework/guidance development team members, and the strategies used to identify and manage them, are clearly described. **(if yes = 3 points)**
**Methods:** This item addresses the use of systematic methods and	a. Systematic and transparent methods were used to identify and review the evidence (e.g., integrated review, scoping review, review of the grey literature, systematic review).	a. Systematic and transparent methods were used to identify and review the evidence. **(if yes = 2 points)**

transparency in reporting; the use of the best available and up-to-date evidence; the consideration of effectiveness and cost-effectiveness of the potential options; and the weighting of benefits and harms in the guidance document.	b. The best available and most contextually relevant evidence was considered.<br>c. The evidence base is current.<br>d. Evidence of effectiveness of the potential options is clearly described, including descriptions of the contexts in which the options were tested.<br>e. Evidence of cost and cost-effectiveness of the potential options is described.<br>f. The weighting of the benefits and harms of the potential options is described.<br>g. There is a link between the recommendations and evidence.<br>h. The rationale behind the recommendations is clear.<br>i. Systematic and transparent methods were used to agree upon the final recommendations (e.g., informal or formal consensus, Delphi method, nominal group methods).	d. Each framework/guidance component and their relationships are clearly described. **(if components were described = 2 points/ + relationship = 3 points)**<br>g. There is a link between the recommendations and evidence. **(if yes = 1 points)**<br>i. Systematic and transparent methods were used to agree upon the final recommendations. **(if yes = 1 points)**
**Recommendations:** This item addresses the outcomes orientation and comprehensiveness of the guidance; the ethical and equity considerations drawn upon in its development; the details for its operationalization; the sociocultural and political alignment of the guidance; and the updating plan.	a. The anticipated outcomes of implementing the recommendations are clearly described (including indicators, performance thresholds or targets, and standards to measure them).<br>b. The recommendations are comprehensive and provide direction to all relevant health system levels (e.g., national, provincial/state), subsystems (e.g., cancer, mental health) and sectors (e.g., primary care, public health).<br>c. The ethical principles used to develop the recommendations are described.<br>d. The recommendations promote equity among the target population (e.g., in terms of age, sex, gender, culture, religion, race, sexual orientation).<br>e. The recommendations' acceptability to, and alignment with, sociocultural and political interests were considered.<br>f. The recommendations are easily identifiable, clear, and succinct.<br>g. The recommendations are actionable and are sufficiently detailed to be operationalized.<br>h. A plan for updating the recommendations is described.	b. The recommendations are comprehensive and provide direction to all relevant health system levels, subsystems, and sectors. **(if yes = 1 points)**<br>c. The ethical principles used to develop the recommendations are described. **(if yes = 1 points)**<br>d. The recommendations promote equity among the target population. **(if yes = 1 points)**<br>e. The recommendations' acceptability to, and alignment with, sociocultural and political interests were considered. **(if yes = 1 points)**<br>f. The recommendations are easily identifiable, clear, and succinct. **(if yes = 1 points)**<br>g. The recommendations are actionable and are sufficiently detailed to be operationalized. **(if yes = 1 points)**<br>h. A plan for updating the recommendations is described. **(if yes = 1 points)**
**Implementability:** This item addresses the barriers and enablers to implementing the recommendations; the	a. Barriers and enablers to the implementation of the recommendations are described, including factors that are internal (e.g., resources, incentives, administrative structure) and external (e.g., legal system, social system, state of the economy, corruption, beliefs) to the health system. A plan to mitigate barriers and optimize enablers is included.	a. Barriers and enablers to the implementation of the recommendations are described, including factors that are internal and external to the health system. A plan to mitigate barriers and optimize enablers is included. **(if yes = 2 points)**

cost and resource considerations in implementing the recommendations; the affordability of implementation and anticipated sustainability of outcomes; the flexibility and transferability of the guidance; and the strategies for disseminating the guidance, monitoring its implementation and evaluating its impact.	b. Cost and resource considerations for the recommended actions are described (e.g., money, time, infrastructure, equipment, administrative capacity, supplies, staffing, and training).<br>c. The stakeholders' acceptability of the recommendations is described.<br>d. The affordability of the recommendations, in the context where implementation will take place, is described.<br>e. The anticipated sustainability and requirements to maintain long-term outcomes is described.<br>f. The recommendations are flexible and there is a description of how they can be adapted or tailored for specific contexts in which they will be implemented.<br>g. A description of the degree to which the recommendations are transferable to other similar or different contexts is provided.<br>h. Strategies for disseminating the health system guidance are described.<br>i. Strategies for assessing the implementation process and the impact of the recommendations are described.	b. Resource considerations for the recommended actions are described. **(if yes = 2 points)**<br>e. The anticipated sustainability and requirements to maintain long-term outcomes is described. **(if yes = 2 points)**<br>g. A description of the degree to which the recommendations are transferable to other similar or different contexts is provided. **(if yes = 1 points)**
**Overall Ranking**	Each item from 1 (lowest quality) to 7 (highest quality)	Each item from 1 (lowest quality) to 7 (highest quality), but we predefined scores for each criterion to improve the reliability.

Reference

Brouwers MC, Lavis JN, Spithoff K, et al. Assessment of health systems guidance using the Appraisal of Guidelines for Research and Evaluation – Health Systems (AGREE-HS) instrument. Health Policy. Jul 2019;123(7):646-651. [doi: 10.1016/j.healthpol.2019.05.004] [Medline: 31160062]

This is a Multimedia Appendix to a full manuscript published in the J Med Internet Res. For full copyright and citation information see https://www.jmir.org/2026/1/e87448

Alami H, Pozelli Sabio R, Pérez EJ, Gagnon MP, Langlois L, Denis JL, Malas K, Rivard L, Salvodelli M, Ag Ahmed MA, Fortin JP
Artificial Intelligence Governance in Health Systems: Systematic Review of Frameworks and Integrative Model Proposal
J Med Internet Res 2026;28:e87448
