# Supplementary material for: Artificial Intelligence Governance in Health Systems: Systematic Review of Frameworks and Integrative Model Proposal
Source: J Med Internet Res. 2026 Jun 8;28:e87448. doi: 10.2196/87448 (PMC13245845; doi:10.2196/87448)
Supplement: Multimedia Appendix 3 [file jmir-v28-e87448-s003.pdf]

Appendix 3. Study characteristics (n=19)

Author, year	Type of docume nt	Study or document objectives	AI governance definition	Study or 1st. author location	Study design or document development process	Study sample or participants in the document development process	Data sources	Data period
WHO, 2024[1]	Guideline	To assist Member States in mapping the benefits and challenges associated with use of Large Multi Modal-models for health and in developing policies and practices for appropriate development, provision and use.	"Governance comprises the steering and rule-making functions of governments and other decision-makers, including international health agencies, for achieving national and global health policy conducive to universal health coverage. Governance is also a political process that involves balancing competing influences and demands… Governance of LMMs, as with overall governance of AI, involves applying current and new legislation and regulations, "soft law" (such as ethical principles), human rights obligations, codes of practice and internal procedures of companies, industry associations and standard-setting bodies."	Switzerland	Consensus process:<br>The Expert Group on the Ethics and Governance of Artificial Intelligence for Health met fortnightly for approximately four months and applied the Consensus Principles and Recommendations from previously issued guidance on the ethics and governance of artificial intelligence for health to the emerging use of LMMs in HS.	20 experts from all WHO regions	The Expert Group first compiled a preliminary mapping of the potential uses and benefits of large multi-modal models, as well as the risks, at the level of the end-user. The Expert Group also identified risks that health systems and societies could encounter with the use of such AI systems. This was supplemented by a comprehensive literature search of: (a) existing and proposed uses of LMMs in healthcare that have materialized over several years of its progressive evolution and use, (b) anticipated uses of LMMs, and (c) critiques and analyses of LMMs that have been published prior to the issuance of this guidance.	Not reported
Morley et al, 2022[2]	Mixed-methods study	To review current health data and AI governance mechanisms being developed or used by Global Digital Health Partnership (GDHP) member countries…, identify commonalities and gaps in approaches,	Not stated	United Kingdom	Mixed methods approach comprising: 1) a rapid scoping review of the academic literature; 2) a systematic review of policy and a thematic analysis of policy documents published by selected GDHP member countries. The findings from this analysis and the literature review were then used to inform and contextualize the following two	1. 32 papers<br>2. Relevant governance documents (ethics, policies, or regulation) from: Australia, Canada, India, Japan, the Republic of Korea, Singapore, the United Kingdom, and the United States.<br>3. The number of semi-structured interviews is not	1. Scopus, PubMed, and Google Scholar.<br>2. Google<br>3. Semi-structured interviews<br>4. Two focus groups	2018-<br>Not reported

		identify examples of best practices, and understand the rationale for policies.			steps: 3) semi-structured interviews and 4) focus group.	reported. The authors only stated that they interviewed relevant policy makers from Australia, Canada, Hong Kong, Italy, the Kingdom of Saudi Arabia, the Netherlands, the Republic of Korea, Singapore, Uruguay, and Wales.<br>4. Two focus groups: 6 participants with expertise in international health and technology organizations.		
WHO, 2021[3]	Guideline	In this report, WHO identifies core principles to promote the ethical use of AI for health.	"Governance in health covers a range of steering and rule-making functions of governments and other decision-makers, including international health agencies, for the achievement of national health policy objectives conducive to universal health coverage. Governance is also a political process that involves balancing competing influences and demands."	Switzerland	Consensus process - Not described	This guidance document was produced jointly by WHO's Health Ethics and Governance unit in the department of Research for Health and by the department of Digital Health and Innovation, is based on the collective views of a WHO Expert Group on Ethics and Governance of AI for Health, which comprised 20 experts in public health, medicine, law, human rights, technology and ethics.	Not reported	Not reported
Reddy et al, 2020[4]	Conceptual paper	To propose a governance model that aims to not only address the ethical and regulatory issues that arise out of the application of AI in healthcare but also stimulate further discussion about governance of AI in healthcare.	Not stated	Australia	Not reported	Not reported	Not reported	Not reported
Jaremko et al, 2019[5]	White paper	To provide a conceptual framework for further discussion and make recommendations	Not stated	Canada	Not reported	Not reported	Not reported	Not reported

		regarding integration of AI in radiology.						
Parker et al, 2024[6]	White paper	To better understand motivations and processes of AI governance in HS in six locations across the United States.	"AI governance is the practice of reviewing, assessing, and evaluating individual AI tools to ensure that they can be used safely, responsibly, fairly, and effectively with the health system's patient population and in compliance with applicable laws."	USA	Not reported	Working group composed by 12 experts from six health systems located across the United States who have established AI governance systems in the past several years.<br>24 experts that participated in a virtual workshop, and multiple other health system representatives.	Interviews and discussions	Unclear (June 2024)
AAAiH, 2023[7]	Report	The Roadmap provides 16 recommendations across 5 priority areas.	Not stated	Australia	Not reported	The Roadmap has been developed through extensive stakeholder engagement across the healthcare sector, including government, industry, peak bodies and consumers. In 2023, faced with a rapid change in technology, further community consultations were undertaken as well as a policy development workshop conducted with representatives of over 30 peak organizations.	Unclear	Not reported
Solaiman 2025[8]	Conceptual paper	To propose comprehensive AI regulation across its entire lifecycle in the health care sector, with a particular focus on patient safety and compliance with legal, ethical, and safety standards	Not stated	Qatar	The Total Lifecycle Approach (TLA) "first emerged when exploring governance models for AI in health care in Qatar, the UAE, and Saudi Arabia. A separate analysis revealed that each country has had important developments in each area of the AI lifecycle, and it was observed that bringing those separate developments together would represent the TLA that could offer a global governance model for AI in health care"	R&D lifecycle phase: Qatar's *Research Guidelines for Healthcare AI Development*<br><br>AI systems approval (market approval) lifecycle phase: Saudi Food & Drug Authority's *Guidance on Artificial Intelligence and Machine Learning technologies based Medical Devices* ('MDS-G010")<br><br>Post implementation lifecycle phase: Abu Dhabi Dep't of Health's *Policy on Use of AI in the Healthcare Sector of the Emirate of Abu Dhabi 5*	Not reported	Not reported

						and Dubai Health Authority's *AI in the Healthcare 2* policy		
Arnaout et al, 2024[9]	Conceptual paper	To establish clinical governance for the responsible deployment of AI tools in healthcare.	Not stated	Canada	Not reported	Not reported	Not reported	Not reported
Whittaker et al, 2023[10]	Mixed-methods study	To describe the establishment of comprehensive governance over the development and implementation of AI tools within our health service.	Not stated	New Zealand (NZ)	Mixed methods approach comprising: 1) background research on international guidance and best practices; 2) surveys and scenario based in depth interviews; 3) analysis of internal data and software systems; 4) integration with Māori data sovereignty principles, and establishment of a representative governance group. The framework was further tested and refined by reviewing AI proposals.	1) 12 documents<br>2) Inpatients and outpatients (n=1377); 12 in-depth interviews.<br>3) Not reported.<br>4) Unclear.	1) WHO, NHSX, Topol, Te Mana Rarauga, NEAC, Algorithm Charter Aotearoa and StatsNZ, Office of the Privacy Commisioner and StatsNZ, Social Wellbeing Agency.<br>2) Cross-sectional survey and in-depth interviews<br>3) Database and software management systems.	Not reported
Carter et al, 2024[11]	Participatory study	To report the first nationally representative deliberative democratic process for developing general recommendations about the use of AI in healthcare.	Not stated	Australia	The procedure followed six core steps for deliberative processes: 1) understanding purpose; 2) relationship building; 3) skill development; 4) information inputs; 5) group dialogue and deliberation; 6) group decision making; and 7) closing. All jurors provided input through iterative cycles of plenary feedback, re-drafting, and voting.	Thirty Australian adults recruited by Sortition Foundation using random invitation and stratified selection to reflect population proportions by gender, age, ancestry, highest level of education, and residential location (state/territory; urban, regional, rural).	Working group discussions	16 March – 2 April 2023
Liao et al, 2022[12]	Case study	To describe the development and nature of governance of clinical AI applications at our institution.	Not stated	USA	Not reported	University of Wisconsin Health (UWH)	This case study did not rely on specific data sets.	Not reported
Bedoya et al, 2022[13]	Conceptual paper	To describe a governance framework that combines current regulatory best practices and	Not stated	USA	Not reported	Duke University Health System	Not reported	Not reported

		lifecycle management of predictive models being used for clinical care.						
Hassan et al, 2025[14]	Conceptual paper	To accomplish the following: define AI and AI governance in healthcare; outline who should be involved in AI governance; describe the key aspects of AI governance that need to be reviewed; describe some of the issues and challenges that need to be considered when adopting AI; and propose an AI governance framework.	"AI governance as an entity comprising of processes, guidelines, and specialized personnel, or subject matter experts dedicated to guiding the ideation, development, deployment, and continuous monitoring of trustworthy and beneficial AI systems. A structure that integrates critical principles, including ethics, fairness, transparency, accountability, and safety, to ensure that AI systems are evaluated and implemented responsibly as well as align with organizational and societal standards."	Canada	Not reported	Not reported	The framework was developed based on the results and analysis of factors that emerged from the authors' previous review of barriers and facilitators of AI in healthcare	Not reported
Economou-Zavlanos et al, 2024[15]	Guideline	To describe a guide and its application to promoting clinical benefit, safety, and equitable impact of algorithmic technologies deployed at author's institution.	Not stated	USA	Not reported	Duke University Health System	This manuscript did not involve analysis or acquisition of datasets.	Not reported
Daye et al, 2022[16]	Conceptual paper	To establish an AI governance structure to ensure appropriate oversight of AI implementation, maintenance, and monitoring in clinical imaging.	"AI governance structures provide mechanisms to decide which tools should be deployed locally and how to best allocate institutional and/or departmental resources to support the clinical implementation of the most valuable and highest-impact applications to improve patient care."	USA	Not reported	Not reported	Not reported	Not reported

Kim et al, 2023[17]	Qualitative study	To describe early-stage research undertaken to support a multi-organizational effort to promote the safe, effective, and equitable adoption of AI software by HS in the United States context specifically.	Not stated	USA	Usability-testing sessions were conducted to design scaffolding for AI adoption standards. First, an interview guides were designed to walk participants through four stages of AI adoption: (1) problem identification and procurement, (2) development and adaptation, (3) clinical integration, and (4) lifecycle management. All interviews were conducted via Zoom by 1-4 project leaders. All interviews were conducted with a single interviewee, except for two sessions where 2 and 4 participants from the same organization participated in each. Each interview was about an hour long, ranging from 34 to 82 minutes.<br>A modified grounded Theory was used to analyze the data.	89 professionals in healthcare and other relevant fields	Semi-structured interviews	July – August 2022
Apfelbacher et al, 2024[18]	Qualitative study	To provide a guidance for the implementation and operational use of AI systems in hospitals.	Not stated	Germany	Explorative qualitative study. To increase engagement, the expert interviews were combined with a role-playing experiment, inspired by Sader. Therefore, the experts were asked to assume specific roles and answer interview questions from that perspective.	Seven stakeholders from different disciplines at UKER and the University Hospital Halle (Saale).	Semi-structured interviews	May 10 and June 28 2023
Kim et al, 2026[19]	Conceptual paper	To introduce "the scalable People, Process, Technology, and Operations (PPTO) framework – adapted from the People, Process, Technology (PPT)_ model".	"a system of rules, practices, processes, and technological tools designed to ensure the responsible development, deployment, and use of AI technologies."	USA	The prototype was "developed through a combination of literature review, analysis of an existing AI governance framework, and key informant interviews". The prototype was then presented during unstructured interviews to collect feedback on requirements and gaps. The feedback was then "used to refine and improve the prototype".	70 health system leaders in the USA "across clinical, technical, operational, and regulatory roles"<br><br>10 key informants "outside healthcare organizations, who have expertise in responsible AI, including bias, ethics, community engagement, organizational behavior, regulation, and credentialing	In-depth interviews and unstructured interviews	Not reported

						Insights gathered through real-world implementation at Duke Health		

This is a Multimedia Appendix to a full manuscript published in the J Med Internet Res. For full copyright and citation information see https://www.jmir.org/2026/1/e87448

Alami H, Pozelli Sabio R, Pérez EJ, Gagnon MP, Langlois L, Denis JL, Malas K, Rivard L, Salvodelli M, Ag Ahmed MA, Fortin JP
Artificial Intelligence Governance in Health Systems: Systematic Review of Frameworks and Integrative Model Proposal
J Med Internet Res 2026;28:e87448

References

1. World Health Organization. Ethics and Governance of Artificial Intelligence for Health: Guidance on Large Multi-Modal Models. World Health Organization; 2024; Available from: https://www.who.int/publications/i/item/9789240084759.
2. Morley J, Murphy L, Mishra A, Joshi I, Karpathakis K. Governing data and artificial intelligence for health care: developing an international understanding. JMIR Form Res. 2022 Jan;6(1):e31623. PMID: WOS:000854067700049. doi: 10.2196/31623.
3. World Health Organization. Ethics and governance of artificial intelligence for health: WHO guidance. World Health Organization; 2021 [Accessed 2025-01-11]; Available from: https://www.who.int/publications/i/item/9789240029200.
4. Reddy S, Allan S, Coghlan S, Cooper P. A governance model for the application of AI in health care. J Am Med Inform Assoc. 2020 Mar 1;27(3):491-7. PMID: 31682262. doi: 10.1093/jamia/ocz192.
5. Jaremko J, Azar M, Bromwich R, Lum A, Alicia Cheong L, Gibert M, et al. Canadian Association of Radiologists White Paper on ethical and legal issues related to artificial intelligence in radiology. Can Assoc Radiol J May 2019;70(2):107-18. doi: 10.1016/j.carj.2019.03.001.
6. Parker V, Economou-Zavlanos N, Silcox C. AI governance in health systems: aligning innovation, accountability and trust. Duke Health; 2024 [Accessed 2025-01-11]; Available from: https://healthaigovernance.duke.edu/news/white-paper-ai-governance-health-systems-aligning-innovation-accountability-and-trust.
7. AAAiH. A national policy roadmap for artificial intelligence in healthcare. AAAiH; 2023 [Accessed 2025-05-05]; Available from: https://aihealthalliance.org/wp-content/uploads/2023/11/AAAiH_NationalPolicyRoadmap_FINAL.pdf.

8. Solaiman B. From bench to bedside: governing health care artificial intelligence (AI) through a “true lifecycle approach”. American Journal of Law & Medicine. 2025;51(3-4):452-78. doi: 10.1017/amj.2025.10091.
9. Arnaout A, Gill P, Virani A, Flatt A, Prodan-Balla N, Byres D, et al. Shaping the future of healthcare in British Columbia: Establishing provincial clinical governance for responsible deployment of artificial intelligence tools. Healthc Manage Forum. 2024;37(5):320-8. doi: 10.1177/08404704241264819.
10. Whittaker R, Dobson R, Jin CK, Style R, Jayathissa P, Hiini K, et al. An example of governance for AI in health services from Aotearoa New Zealand. NPJ Digit Med. 2023;6(1). doi: 10.1038/s41746-023-00882-z.
11. Carter S, Aquino Y, Carolan L, Frost E, Degeling C, Rogers W, et al. How should artificial intelligence be used in Australian health care? Recommendations from a citizens’ jury. Med J Aust. 2024;220(8):409-16. doi: 10.5694/mja2.52283.
12. Liao F, Adelaine S, Afshar M, Patterson B. Governance of clinical AI applications to facilitate safe and equitable deployment in a large health system: key elements and early successes. Front Digit Health. 2022;4:931439. PMID: 36093386. doi: 10.3389/fdgth.2022.931439.
13. Bedoya A, Economou-Zavlanos N, Goldstein B, Young A, Jelovsek J, O'Brien C, et al. A framework for the oversight and local deployment of safe and high-quality prediction models. J Am Med Inform Assoc. 2022;29(9):1631-36. doi: 10.1093/jamia/ocac078.
14. Hassan M, Borycki E, Kushniruk A. Artificial intelligence governance framework for healthcare. Healthc Manage Forum. 2025 Mar;38(2):125-30. PMID: 39470044. doi: 10.1177/08404704241291226.
15. Economou-Zavlanos N, Bessias S, Cary M, Bedoya A, Goldstein B, Jelovsek J, et al. Translating ethical and quality principles for the effective, safe and fair development, deployment and use of artificial intelligence technologies in healthcare. J Am Med Inform Assoc. 2024;31(3):705-13. doi: 10.1093/jamia/ocad221.
16. Daye D, Wiggins W, Lungren M, Alkasab T, Kottler N, Allen B, et al. Implementation of clinical artificial intelligence in radiology: who decides and how? Radiology. 2022 Dec;305(3):555-63. PMID: 35916673. doi: 10.1148/radiol.212151.
17. Kim J, Boag W, Gulamali F, Hasan A, Hogg H, Lifson M, et al. Organizational governance of emerging technologies: AI adoption in healthcare. Presented at: FAccT ’23: Proceedings of the 2023 ACM Conference on Fairness, Accountability, and Transparency; Chicago, IL, USA: Association for Computing Machinery; Jun 12-15, 2023. p. 1396–417.
18. Apfelbacher T, Kocman SE, Prokosch HU, Christoph J. A governance framework for the implementation and operation of AI applications in a university hospital. Stud Health Technol Inform. 2024 Aug 22;316:776-80. PMID: 39176908. doi: 10.3233/SHTI240527.
19. Kim JY, Hasan A, Balu S, Sendak M. People process technology and operations framework for establishing AI governance in healthcare organizations. npj Digital Medicine. 2026;9(1). doi: 10.1038/s41746-026-02419-6.
