# Supplementary material for: Artificial Intelligence Governance in Health Systems: Systematic Review of Frameworks and Integrative Model Proposal
Source: J Med Internet Res. 2026 Jun 8;28:e87448. doi: 10.2196/87448 (PMC13245845; doi:10.2196/87448)
Supplement: Multimedia Appendix 4 [file jmir-v28-e87448-s004.pdf]

Appendix 4. Characteristics of the AI governance frameworks for HS

Author/ year	Aim/purpose	Framework name	Source	Target audience	Main components of the framework
WHO, 2024[1]	"To assist Member States in mapping the benefits and challenges associated with use of Large Multi Modal-models for health and in developing policies and practices for appropriate development, provision and use."	Ethics and governance of large multimodal models in healthcare and medicine	Academic based - Practitioners	Governments, developers, companies	1. Design and development<br>2. Provision phase<br>3. Deployment phase
Morley et al, 2022[2]	"To review current health data and AI governance mechanisms being developed or used by Global Digital Health Partnership (GDHP) member countries…, identify commonalities and gaps in approaches, identify examples of best practices, and understand the rationale for policies."	N/A	Academic	Governments	1. Business and use case development<br>2. Design phase<br>3. Training and test data procurement<br>4. Building<br>5. Testing and validation<br>6. Deployment<br>7. Monitoring
WHO, 2021[3]	"In this report, WHO identifies core principles to promote the ethical use of AI for health."	Framework for governance of artificial intelligence for health	Academic based - Practitioners	Ministries, Regulatory agencies, stakeholders through the HS, technologists and software developers, companies, universities, medical associations, international organizations	1. Governance of data<br>2. Control and benefit sharing<br>3. Governance of the private sector<br>4. Governance of the public sector<br>5. Regulatory considerations<br>6. Policy observatory and model legislation<br>7. Global governance of AI
Reddy et al, 2020[4]	"To propose a governance model that aims to not only address the ethical and regulatory issues that arise out of the application of AI in healthcare but also stimulate further discussion about governance of AI in healthcare."	Governance Model for AI in Healthcare (GMAIH)	Academic	Governmental bodies	1. Fairness<br>2. Transparency<br>3. Trustworthiness<br>4. Accountability
Jaremko et al, 2019[5]	"To provide a conceptual framework for further discussion and make recommendations regarding integration of AI in radiology"	N/A	Academic based - Practitioners	Canadian Association of Radiologists (CAR), Radiologists	1. Data privacy<br>2. Technical aspects of implementing data privacy<br>3. Role of data custodian - data sharing<br>4. Role of radiologist - liability
Parker et al, 2024[6]	"To better understand motivations and processes" of AI governance in HS in six locations across the United States	N/A	Academic - based Practitioners	Decision-makers, HS, developers and other stakeholders (clinical societies, clinical professional schools, etc.)	1. Organizational alignment and engagement<br>2. Identification and registration of AI tools<br>3. Review and assessment<br>4. Monitoring and surveillance
AAAiH, 2023[7]	"The Roadmap provides 16 recommendations across 5 priority areas"	AI in Healthcare National Policy Roadmap	Academic based - Practitioners	Government and decision-makers in the Australian context	1. AI safety, quality, ethics and security<br>2. Workforce<br>3. Consumers<br>4. Industry<br>5. Research

Solaiman 2025[8]	To propose comprehensive AI regulation across its entire lifecycle in the health care sector, with a particular focus on patient safety and compliance with legal, ethical, and safety standards	The True Lifecycle Approach towards governing AI in health care	Academic based - Practitioners	Researchers and developers, regulatory approval authorities, and national health authorities	1. Research and development (informed consent; data confidentiality; liability risk assessment)<br>2. AI systems approval (regulatory compliance; safety & efficacy; consent protocols)<br>3. Post-implementation governance (ongoing monitoring; liability; patient rights; data protection)
Arnaout et al, 2024[9]	“To establish clinical governance for the responsible deployment of AI tools in healthcare.”	N/A	Practitioners	Health system authorities	1. Clinical need and context<br>2. Ethics<br>3. Risk assessment and management<br>4. Data<br>5. Clear, transparent communication to patients, communities, and providers<br>6. Healthcare workforce training<br>7. Ongoing surveillance<br>8. Success metrics
Whittaker et al, 2023[10]	“To describe the establishment of comprehensive governance over the development and implementation of AI tools within our health service.”	N/A	Practitioners	Healthcare organizations, developers, Aoteroa NZ health service	1. Consumer<br>2. Mâori<br>3. Equity<br>4. Ethics<br>5. Clinical<br>6. Data<br>7. Technical<br>8. Legal
Carter et al, 2024[11]	“To report the first nationally representative deliberative democratic process for developing general recommendations about the use of AI in healthcare.”	N/A	Citizens	AI researchers, clinicians, developers, health service users, decision-makers	1. The need for an overarching, independently governed charter and framework<br>2. Balancing benefits and harms<br>3. Fairness and bias<br>4. Patients’ rights and choices<br>5. Clinical governance and training<br>6. Technical governance and standards<br>7. Data governance and use<br>8. Open-source software<br>9. AI evaluation and assessment<br>10. Education and communication
Liao et al, 2022[12]	“To describe the development and nature of governance of clinical AI applications at our institution.”	N/A	Academic - based Practitioners	University hospitals	1. Clinical<br>2. Operational<br>3. Leadership
Bedoya et al, 2022[13]	“To describe a governance framework that combines current regulatory best practices and lifecycle management of predictive models being used for clinical care.”	Algorithm- Based Clinical Decision Support (ABCDS)	Academic	“Clinicians, data scientists, and executive leadership from hospitals and HS”	The framework encompasses the full lifecycle of an algorithm or model divided into 4 stages, as well as 3 checkpoints distributed across the stages. Stages of the lifecycle:<br><br>1. Model development<br>2. Silent evaluation

					3. Effectiveness evaluation<br>4. General deployment
Hassan et al, 2025[14]	"To accomplish the following: define AI and AI governance in healthcare; outline who should be involved in AI governance; describe the key aspects of AI governance that need to be reviewed; describe some of the issues and challenges that need to be considered when adopting AI; and propose an AI governance framework."	N/A	Academic	Healthcare organizations	An adoption-centred governance framework that covers the entire cycle of an AI system and is organized around 5 main components:<br><br>1. Provide oversight on end-to-end process of AI<br>2. Identify all entities associated with each of the oversight domains<br>3. Based on the factors impacting adoption<br>4. Embed processes to develop assessment tools and repositories for collecting evidence to support adoption barriers<br>5. Apply one or more validated implementation, evaluation, and long-term use frameworks
Economou-Zavlanos et al, 2024[15]	"To describe a guide and its application to promoting clinical benefit, safety, and equitable impact of algorithmic technologies deployed at author's institution."	The ABCDS Oversight Implementation Guide	Academic based - Practitioners	HS leadership, "stakeholders engaged in developing and deploying algorithmic technologies"	1. Clinical value and safety<br>2. Fairness and equity<br>3. Usability and adoption<br>4. Regulatory compliance<br>5. Transparency and accountability
Daye et al, 2022[16]	"To establish an AI governance structure to ensure appropriate oversight of AI implementation, maintenance, and monitoring in clinical imaging."	AI Governance Road Map	Not stated	Organizational leaders on clinical imaging settings	The framework is designed around the following questions:<br><br>1. Who decides which tools to implement?<br>2. What factors should be considered when assessing an application for implementation?<br>3. How should applications be implemented in clinical practice?<br>4. How should tools be monitored and maintained after clinical implementation?
Kim et al, 2023[17]	"To describe early-stage research undertaken to support a multi-organizational effort to promote the safe, effective, and equitable adoption of AI software by HS in the United States context specifically."	N/A	Academic based - Practitioners	Algorithm developers, organizational leaders, decision-makers, regulated enterprises	1. Identify and prioritize a problem<br>2. Identify requirements for an AI product as a viable component of the solution<br>3. Develop measures of outcomes and success of the AI product<br>4. Design a new optimal workflow to facilitate integration<br>5. Evaluate safety, effectiveness, and equity concerns of the AI product in the intended setting prior to clinical use<br>6. Execute AI product rollout, workflow integration, communication, education, and scaling<br>7. After operationalization, monitor and maintain the AI product and impacted ecosystem

					8. Update or decommission the AI product and impacted ecosystem
Apfelbacher et al, 2024[18]	"To provide a guidance for the implementation and operational use of AI systems in hospitals."	N/A	Academic	Stakeholders engaged in the implementation of AI in University hospitals	1. Before the implementation<br>2. During the implementation<br>3. After the implementation
Kim et al, 2026[19]	"To guide governance across key domains" and offer "a systematic roadmap for safe, effective, and equitable AI adoption in" healthcare organizations	People, Process, Technology, and Operations (PPTO) Framework	Academic based - Practitioners	Healthcare delivery organizations	1. People: governance committee comprised of 4 subcommittees – Implementation and Monitoring; Quantitative assessment; Ethics and Legal; Operation<br>2. Process: systematic process to govern AI across the lifecycle, "guiding the governance committee in oversight and decision-making while informing project teams as their AI initiatives progress through each stage"<br>3. Technology: "AI DevOPs approach across the Development and Adaptation, Clinical Integration, and Lifecycle Management stages for the governance committee to oversee validation and monitoring processes"<br>4. Operation: Executive sponsorship, budgeting, success measures, metrics, and capabilities

This is a Multimedia Appendix to a full manuscript published in the J Med Internet Res. For full copyright and citation information see https://www.jmir.org/2026/1/e87448

Alami H, Pozelli Sabio R, Pérez EJ, Gagnon MP, Langlois L, Denis JL, Malas K, Rivard L, Salvodelli M, Ag Ahmed MA, Fortin JP
Artificial Intelligence Governance in Health Systems: Systematic Review of Frameworks and Integrative Model Proposal
J Med Internet Res 2026;28:e87448

References

1. World Health Organization. Ethics and Governance of Artificial Intelligence for Health: Guidance on Large Multi-Modal Models. World Health Organization; 2024; Available from: https://www.who.int/publications/i/item/9789240084759.
2. Morley J, Murphy L, Mishra A, Joshi I, Karpathakis K. Governing data and artificial intelligence for health care: developing an international understanding. JMIR Form Res. 2022 Jan;6(1):e31623. PMID: WOS:000854067700049. doi: 10.2196/31623.

3. World Health Organization. Ethics and governance of artificial intelligence for health: WHO guidance. World Health Organization; 2021 [Accessed 2025-01-11]; Available from: https://www.who.int/publications/i/item/9789240029200.
4. Reddy S, Allan S, Coghlan S, Cooper P. A governance model for the application of AI in health care. J Am Med Inform Assoc. 2020 Mar 1;27(3):491-7. PMID: 31682262. doi: 10.1093/jamia/ocz192.
5. Jaremko J, Azar M, Bromwich R, Lum A, Alicia Cheong L, Gibert M, et al. Canadian Association of Radiologists White Paper on ethical and legal issues related to artificial intelligence in radiology. Can Assoc Radiol J May 2019;70(2):107-18. doi: 10.1016/j.carj.2019.03.001.
6. Parker V, Economou-Zavlanos N, Silcox C. AI governance in health systems: aligning innovation, accountability and trust. Duke Health; 2024 [Accessed 2025-01-11]; Available from: https://healthaigovernance.duke.edu/news/white-paper-ai-governance-health-systems-aligning-innovation-accountability-and-trust.
7. AAAiH. A national policy roadmap for artificial intelligence in healthcare. AAAiH; 2023 [Accessed 2025-05-05]; Available from: https://aihealthalliance.org/wp-content/uploads/2023/11/AAAiH_NationalPolicyRoadmap_FINAL.pdf.
8. Solaiman B. From bench to bedside: governing health care artificial intelligence (AI) through a "true lifecycle approach". American Journal of Law & Medicine. 2025;51(3-4):452-78. doi: 10.1017/amj.2025.10091.
9. Arnaout A, Gill P, Virani A, Flatt A, Prodan-Balla N, Byres D, et al. Shaping the future of healthcare in British Columbia: Establishing provincial clinical governance for responsible deployment of artificial intelligence tools. Healthc Manage Forum. 2024;37(5):320-8. doi: 10.1177/08404704241264819.
10. Whittaker R, Dobson R, Jin CK, Style R, Jayathissa P, Hiini K, et al. An example of governance for AI in health services from Aotearoa New Zealand. NPJ Digit Med. 2023;6(1). doi: 10.1038/s41746-023-00882-z.
11. Carter S, Aquino Y, Carolan L, Frost E, Degeling C, Rogers W, et al. How should artificial intelligence be used in Australian health care? Recommendations from a citizens' jury. Med J Aust. 2024;220(8):409-16. doi: 10.5694/mja2.52283.
12. Liao F, Adelaine S, Afshar M, Patterson B. Governance of clinical AI applications to facilitate safe and equitable deployment in a large health system: key elements and early successes. Front Digit Health. 2022;4:931439. PMID: 36093386. doi: 10.3389/fdgth.2022.931439.
13. Bedoya A, Economou-Zavlanos N, Goldstein B, Young A, Jelovsek J, O'Brien C, et al. A framework for the oversight and local deployment of safe and high-quality prediction models. J Am Med Inform Assoc. 2022;29(9):1631-36. doi: 10.1093/jamia/ocac078.
14. Hassan M, Borycki E, Kushniruk A. Artificial intelligence governance framework for healthcare. Healthc Manage Forum. 2025 Mar;38(2):125-30. PMID: 39470044. doi: 10.1177/08404704241291226.
15. Economou-Zavlanos N, Bessias S, Cary M, Bedoya A, Goldstein B, Jelovsek J, et al. Translating ethical and quality principles for the effective, safe and fair development, deployment and use of artificial intelligence technologies in healthcare. J Am Med Inform Assoc. 2024;31(3):705-13. doi: 10.1093/jamia/ocad221.

16. Daye D, Wiggins W, Lungren M, Alkasab T, Kottler N, Allen B, et al. Implementation of clinical artificial intelligence in radiology: who decides and how? Radiology. 2022 Dec;305(3):555-63. PMID: 35916673. doi: 10.1148/radiol.212151.
17. Kim J, Boag W, Gulamali F, Hasan A, Hogg H, Lifson M, et al. Organizational governance of emerging technologies: AI adoption in healthcare. Presented at: FAccT '23: Proceedings of the 2023 ACM Conference on Fairness, Accountability, and Transparency; Chicago, IL, USA: Association for Computing Machinery; Jun 12-15, 2023. p. 1396–417.
18. Apfelbacher T, Kocman SE, Prokosch HU, Christoph J. A governance framework for the implementation and operation of AI applications in a university hospital. Stud Health Technol Inform. 2024 Aug 22;316:776-80. PMID: 39176908. doi: 10.3233/SHTI240527.
19. Kim JY, Hasan A, Balu S, Sendak M. People process technology and operations framework for establishing AI governance in healthcare organizations. npj Digital Medicine. 2026;9(1). doi: 10.1038/s41746-026-02419-6.
