# Supplementary material for: Artificial Intelligence Governance in Health Systems: Systematic Review of Frameworks and Integrative Model Proposal
Source: J Med Internet Res. 2026 Jun 8;28:e87448. doi: 10.2196/87448 (PMC13245845; doi:10.2196/87448)
Supplement: Multimedia Appendix 5 [file jmir-v28-e87448-s005.pdf]

Appendix 5. AI governance structure functions identified across the included frameworks (n=19)

**Author/Year**	**Governance structure functions by level**
International level	
WHO, 2024[1]	• Avoiding a 'race to the bottom' among companies seeking a first-mover advantage in which standards of safety and efficacy are ignored, and among governments seeking advantage in the geopolitical race for technological supremacy.<br>• Ensuring that all companies meet minimum standards of safety and efficacy.<br>• Avoiding introduction of regulations that provide a competitive advantage or disadvantage for either companies or governments.<br>• Making governments accountable for their investments and participation in the development and deployment of AI-based systems and ensure that they introduce appropriate regulations that respect ethical principles, human rights, and international law.
Morley et al, 2022[2]	• International leadership could help alleviate government nervousness about public–private partnerships by supporting mechanisms for external scrutiny of private industry partners, standardizing terms for sharing and accessing patient data, and securing fair commercial terms between public and private partners.<br>• A means of protecting the interests of public HS faced with increasing involvement from private technology companies.
WHO, 2021[3]	• Steering and rule-making functions of governments and other decision-makers for the achievement of national health policy objectives conducive to universal health coverage.<br>• Ensuring that all governments can adapt to changes brought by these technologies as they become ever more sophisticated and powerful.
National level	
Reddy et al, 2020[4]	• Addressing ethical, quality, and regulatory and safety concerns.
Jaremko et al, 2019[5]	• Developing guidelines for appropriate deployment of AI assistive tools in hospital departments and radiology groups, seeking to minimize potential harm and institutional liability for malpractice in case of medical error involving AI.
Parker et al, 2024[6]	• Evaluating AI tools to be used in the health system.<br>• Ensuring that these tools are deployed safely, ethically, and in compliance with regulatory standards.<br>• Protecting patient safety and fostering trust.<br>• Facilitating innovation by providing clear guidelines and processes for assessing and implementing AI-based technologies.
AAAiH, 2023[7]	• *Not stated*

Solaiman 2025[8]	• Addressing current regulatory gaps with a particular focus on ethical and patient safety concerns.<br>• Considering health care law, ethics, and patient safety throughout the full AI lifecycle, including research and development, market approval, and post-implementation.
Local level	
Arnaout et al, 2024[9]	• Identifying clear clinical pain points or gaps which an AI solution may address.<br>• Establishing clear guiding principles/standards for responsible AI use, informed by strong ethical, risk, legal, cultural humility, and safety frameworks, comply with policies and regulations, and establish robust measures to safeguard sensitive patient data.<br>• Establishing a streamlined central intake, assessment, and an equity-driven prioritization process for resourcing AI opportunities.<br>• Prioritizing and coordinating AI solution implementation activities.<br>• Ensuring economies of scale, avoidance of duplication, and creation of efficiency for large scale implementation of AI tools, while still creating space for local innovation in model development.<br>• Providing oversight for post-implementation evaluation metrics, including iterative safety and clinical outcomes, as well as adoption metrics.
Whittaker et al, 2023[10]	• Ensuring that the relevant areas for AI development or implementation are systematically reviewed and potential risks are identified.<br>• Providing oversight about appropriateness, safety, effectiveness, ethics, and ongoing improvement of any AI research, development, projects, partnerships, contracts, or implementation at Waitematā.
Carter et al, 2024[11]	• Ensuring an ethical and moral pathway to the development and deployment of AI in health diagnosis and detection.
Organizational level	
Liao et al, 2022[12]	• Establishing a full lifecycle for models, including processes for evaluation and potential adoption of models, monitoring to ensure they continuously meet the needs of all constituents, and appropriate processes for periodic revaluation and decommissioning of models no longer needed or not functioning correctly.
Bedoya et al, 2022[13]	• Establishing a rigorous governance structure to provide support at 3 checkpoints between these phases, each featuring review by an expert multidisciplinary subcommittee.
Hassan et al, 2025[14]	• Supporting the development, use, and sustainability of trustworthy AI systems.<br>• Ensures oversight over the barriers identified and provides a structured mechanism to allow for implementation of trustworthy AI.<br>• Ensure the safe and trustworthy implementation of AI systems.<br>• Providing guidance on regulatory, legal, data governance, bias, ethics, risk, accountability, and funding to enable successful implementation and adoption of AI systems.
Economou-Zavlanos et al, 2024[15]	• Ensuring algorithmic technologies is based on 5 guiding principles applied across the development lifecycle: (1) transparency and accountability; (2) clinical value and safety; (3) fairness and equity; (4) usability and adoption; and (5) regulatory compliance.

Daye et al, 2022[16]	• Guide the evaluation, selection, procurement, implementation, and ongoing support for internally developed and vendor-supplied solutions.<br>• Balancing the desire to drive innovation forward with processes to ensure quality and safety in clinical implementation.<br>• Establishing processes to stratify risk and determine the appropriate path for initial implementation and monitoring.
Kim et al, 2023[17]	• *Not stated*
Apfelbacher et al, 2024[18]	• *Not stated*
Kim et al, 2026[19]	• Ensuring effective oversight and management of AI initiatives within healthcare organizations.<br>• Providing guidance and actionable strategies to AI development teams throughout problem identification, development and adaptation, clinical integration, and lifecycle management.

This is a Multimedia Appendix to a full manuscript published in the J Med Internet Res. For full copyright and citation information see https://www.jmir.org/2026/1/e87448

Alami H, Pozelli Sabio R, Pérez EJ, Gagnon MP, Langlois L, Denis JL, Malas K, Rivard L, Salvodelli M, Ag Ahmed MA, Fortin JP
Artificial Intelligence Governance in Health Systems: Systematic Review of Frameworks and Integrative Model Proposal
J Med Internet Res 2026;28:e87448

References

1. World Health Organization. Ethics and Governance of Artificial Intelligence for Health: Guidance on Large Multi-Modal Models. World Health Organization; 2024; Available from: https://www.who.int/publications/i/item/9789240084759.
2. Morley J, Murphy L, Mishra A, Joshi I, Karpathakis K. Governing data and artificial intelligence for health care: developing an international understanding. JMIR Form Res. 2022 Jan;6(1):e31623. PMID: WOS:000854067700049. doi: 10.2196/31623.
3. World Health Organization. Ethics and governance of artificial intelligence for health: WHO guidance. World Health Organization; 2021 [Accessed 2025-01-11]; Available from: https://www.who.int/publications/i/item/9789240029200.
4. Reddy S, Allan S, Coghlan S, Cooper P. A governance model for the application of AI in health care. J Am Med Inform Assoc. 2020 Mar 1;27(3):491-7. PMID: 31682262. doi: 10.1093/jamia/ocz192.
5. Jaremko J, Azar M, Bromwich R, Lum A, Alicia Cheong L, Gibert M, et al. Canadian Association of Radiologists White Paper on ethical and legal issues related to artificial intelligence in radiology. Can Assoc Radiol J May 2019;70(2):107-18. doi: 10.1016/j.carj.2019.03.001.

6. Parker V, Economou-Zavlanos N, Silcox C. AI governance in health systems: aligning innovation, accountability and trust. Duke Health; 2024 [Accessed 2025-01-11]; Available from: https://healthaigovernance.duke.edu/news/white-paper-ai-governance-health-systems-aligning-innovation-accountability-and-trust.
7. AAAiH. A national policy roadmap for artificial intelligence in healthcare. AAAiH; 2023 [Accessed 2025-05-05]; Available from: https://aihealthalliance.org/wp-content/uploads/2023/11/AAAiH_NationalPolicyRoadmap_FINAL.pdf.
8. Solaiman B. From bench to bedside: governing health care artificial intelligence (AI) through a "true lifecycle approach". American Journal of Law & Medicine. 2025;51(3-4):452-78. doi: 10.1017/amj.2025.10091.
9. Arnaout A, Gill P, Virani A, Flatt A, Prodan-Balla N, Byres D, et al. Shaping the future of healthcare in British Columbia: Establishing provincial clinical governance for responsible deployment of artificial intelligence tools. Healthc Manage Forum. 2024;37(5):320-8. doi: 10.1177/08404704241264819.
10. Whittaker R, Dobson R, Jin CK, Style R, Jayathissa P, Hiini K, et al. An example of governance for AI in health services from Aotearoa New Zealand. NPJ Digit Med. 2023;6(1). doi: 10.1038/s41746-023-00882-z.
11. Carter S, Aquino Y, Carolan L, Frost E, Degeling C, Rogers W, et al. How should artificial intelligence be used in Australian health care? Recommendations from a citizens' jury. Med J Aust. 2024;220(8):409-16. doi: 10.5694/mja2.52283.
12. Liao F, Adelaine S, Afshar M, Patterson B. Governance of clinical AI applications to facilitate safe and equitable deployment in a large health system: key elements and early successes. Front Digit Health. 2022;4:931439. PMID: 36093386. doi: 10.3389/fdgth.2022.931439.
13. Bedoya A, Economou-Zavlanos N, Goldstein B, Young A, Jelovsek J, O'Brien C, et al. A framework for the oversight and local deployment of safe and high-quality prediction models. J Am Med Inform Assoc. 2022;29(9):1631-36. doi: 10.1093/jamia/ocac078.
14. Hassan M, Borycki E, Kushniruk A. Artificial intelligence governance framework for healthcare. Healthc Manage Forum. 2025 Mar;38(2):125-30. PMID: 39470044. doi: 10.1177/08404704241291226.
15. Economou-Zavlanos N, Bessias S, Cary M, Bedoya A, Goldstein B, Jelovsek J, et al. Translating ethical and quality principles for the effective, safe and fair development, deployment and use of artificial intelligence technologies in healthcare. J Am Med Inform Assoc. 2024;31(3):705-13. doi: 10.1093/jamia/ocad221.
16. Daye D, Wiggins W, Lungren M, Alkasab T, Kottler N, Allen B, et al. Implementation of clinical artificial intelligence in radiology: who decides and how? Radiology. 2022 Dec;305(3):555-63. PMID: 35916673. doi: 10.1148/radiol.212151.
17. Kim J, Boag W, Gulamali F, Hasan A, Hogg H, Lifson M, et al. Organizational governance of emerging technologies: AI adoption in healthcare. Presented at: FAccT '23: Proceedings of the 2023 ACM Conference on Fairness, Accountability, and Transparency; Chicago, IL, USA: Association for Computing Machinery; Jun 12-15, 2023. p. 1396–417.

18. Apfelbacher T, Kocman SE, Prokosch HU, Christoph J. A governance framework for the implementation and operation of AI applications in a university hospital. Stud Health Technol Inform. 2024 Aug 22;316:776-80. PMID: 39176908. doi: 10.3233/SHTI240527.
19. Kim JY, Hasan A, Balu S, Sendak M. People process technology and operations framework for establishing AI governance in healthcare organizations. npj Digital Medicine. 2026;9(1). doi: 10.1038/s41746-026-02419-6.
