# Supplementary material for: Artificial Intelligence Governance in Health Systems: Systematic Review of Frameworks and Integrative Model Proposal
Source: J Med Internet Res. 2026 Jun 8;28:e87448. doi: 10.2196/87448 (PMC13245845; doi:10.2196/87448)
Supplement: Multimedia Appendix 6 [file jmir-v28-e87448-s006.pdf]

Appendix 6. Actors' roles and responsibilities

Author, year	Actors' roles and responsibilities
WHO, 2024[1]	Developers: The design and development of general-purpose foundation models can introduce serious risks (…). Elimination or mitigation of such risks is the responsibility of the developer (...). At least eight risks should be addressed by the developer (…): Bias (associated with the design and training data); privacy (of training and other input data); labour concerns (outsourced filtering of data to remove offensive content); the carbon and water footprints; false information, hate speech or misinformation; safety and cybersecurity; and preserving the epistemic authority of humans; exclusive control of LMMs<br>Developers: Developers that design an LMM that shall or could be used in health care (…) shall consider ethics certification or training for programmers. This would bring AI developers in line with requirements in the medical profession and increase trust in their products and services; conduct data protection impact assessments to assess the risk that data processing operations would go against the rights and freedom of individuals and its impact on the protection of personal data; Developers should train LMMs on data collected according to best-practice data protection rules; Keep data sets used to train LMMs up to date and appropriate for the contexts in which the system may be used; Ensure transparency about the data used to train a model;<br>Developers should pay data workers a living wage and provide them with mental health services and other forms of counselling. Developers should also introduce safeguards to protect workers from any distress ; Ensure that LMMs are designed not only by scientists and engineers; Ensure that LMMs are designed to perform well-defined tasks with the necessary accuracy and reliability to improve the capacity of health systems and advance patient interests; be able to predict and understand potential secondary outcomes; Design for values based on consensus principles and ethical norms (a paradigm for basing design on the values of human dignity, freedom, equality and solidarity); take all possible steps to reduce energy consumption (such as by improving the energy efficiency of a model).<br>Governments: Update their labour standards to extend benefits to all data workers, to promote a "level playing field" among companies and to ensure that labour standards are maintained and improved over time; Have and enforce strong data protection laws and regulations for the use of health data and protecting individual rights; issue "target product profiles" to delineate preferences and characteristics of LMMs intended for use in health care and medicine, especially if governments anticipate eventual purchase of such tools for use in government-run health

	systems; Design and development standards and requirements: Governments could require developers to ensure that the design and development of a general-purpose foundation model achieve certain outcomes throughout its life cycle. They could include requirements for the predictability of the model and its interpretability, corrigibility, safety and cybersecurity; Conduct audits during early AI development; Require developers (…) to address concern about their carbon and water footprints; require developers to ensure that any deployment of a general-purpose foundation model includes notification and reminders to end-users that the content was generated by a machine and not a human being; Require or create incentives for developers to register early-stage AI algorithms or systems to be used in health care and medicine. Early registration could encourage publication of negative results, prevent publication bias or over-optimistic interpretation of results and facilitate integration of knowledge that benefits patients; Governments should encourage the development of open-source LMMs by requiring that foundation models built with government funding or intellectual property are widely accessible, in the same way that governments have required open access to government-funded research. Governments should support open-source research and development in public facilities, including next-generation models, under controlled conditions, with public oversight<br><br>Governments should, as resources permit, assign an existing or new regulatory agency to assess and approve LMMs and applications intended for use in health care or medicine; Ensure that data protection rules apply to data entered into an LMM or application by a user; Ensure that LMMs and applications used in health care and medicine, irrespective of the risk or benefit associated with the AI technology, meet ethical obligations and human rights standards that affect, for example, a person's dignity, autonomy or privacy; Governments should enact laws and policies that require providers and developers to conduct impact assessments of LMMs and applications, which should address ethics, human rights, safety and data protection, throughout the life cycle of an AI system; Governments should ensure that, for an LMM or application that is regulated as a medical device, the developer and/or provider is responsible for the burden of proof that the device performs as marketed and that it meets the requirements of the country's laws or amended laws; Governments should ensure that LMMs or applications for supporting clinical decisions that are not yet approved for use not be used on an experimental basis outside an authorized clinical trial setting; Governments should use consumer protection laws to ensure that any negative consequences of use of LMMs and applications do not affect users, including patients;<br><br>Governments that supply an LMM or application to a health system could use their procurement authority to foster certain practices among developers, providers and deployers; Governments could hold providers or developers responsible for inaccurate, false or toxic content issued by an LMM after its release, which has not been corrected

	or avoided by either the provider or the developer; Governments should require ongoing operational disclosures by both developers and providers to ensure that LMMs and applications can be used safely; Governments should establish liability along the value chain of the development, provision and deployment of LMMs and applications to ensure that a victim of damage can claim compensation, irrespective of the difficulty of assigning blame and of the responsibilities of the different entities involved in the development and deployment of the technology; Governments should support collective development of international rules for the governance of LMMs and other forms of AI used in health care.<br><br>Governments, subject to independent oversight, could construct infrastructure that is then used by developers to construct LMMs for health care and medicine. (…). As uses of LMMs for health proliferate, development of LMMs that adhere to ethical principles could be encouraged by the provision of not-for-profit or public infrastructure, including computing power and public datasets. Such infrastructure, which could be accessible to developers in the public, private and not-for-profit sectors, could require users to adhere to ethical principles and values in exchange for access. It could also help to avoid exclusive control of an LMM by a developer and "level the playing field" between the largest companies and developers that do not have access to such infrastructure and resources.<br><br>Human oversight colleges: Potential users and all direct and indirect stakeholders, including medical providers, scientific researchers, health-care professionals and patients, should be engaged from the early stages of AI development in structured, inclusive, transparent design and given opportunities to raise ethical issues, voice concerns and provide input for the AI application under consideration. Such input could be provided through “human oversight colleges”.<br><br>Regulatory agencies should introduce legal obligations and establish incentives, such as pre-certification programmes, to require and encourage developers to identify and avoid ethical risks, including bias or undermining autonomy.<br><br>Governments and International agencies should issue “target product profiles” to delineate preferences and characteristics of LMMs intended for use in health care and medicine.<br><br>Deployers: A deployer should use information from developers or providers to decide not to use an LMM or application in an inappropriate setting, because of biases in the training data, contextual bias that renders the LMM inappropriate for the setting or other avoidable errors or potential risks known to the deployer; Deployers should communicate any risks that they should reasonably know could result from use of an LMM and any errors or

	mistakes that have harmed users. (…). In some circumstances, a deployer may be responsible, even if not required by a law or regulation, for suspending use or removing an LMM or application from the market to avoid harm; Deployers can take steps to improve the affordability and accessibility of an LMM. A deployer can ensure that pricing or subscription fees for use of an LMM correspond to the capacity of a government or other user to pay and should ensure that appropriate LMMs are trained and provided in languages and scripts that can be used by people who are otherwise ignored or excluded from the benefits of technology. Deployers should also request providers and developers to ensure that current and future LMMs are available in several languages.<br><br>Ministries of health and universities (health science faculties): should train health-care professionals and clinicians: (i) to understand how LMMs make decisions (and the limits of understanding how such decisions are made), (ii) to identify and understand concerns about appropriate use, (iii) in methods to avoid automation bias, (iv) to engage with and educate patients who may be or are considering use of LMMs, and (v) on the cybersecurity risks associated with use of LMMs.
Morley et al, 2022[2]	The interviewees and focus group participants agreed on the need for an international body responsible for working with national representatives to build capability and ensure the implementation of recommended policies for each phase of the AI life cycle.<br><br>International leadership could help alleviate government nervousness about public–private partnerships by supporting mechanisms for external scrutiny of private industry partners, standardizing terms for sharing and accessing patient data, and securing fair commercial terms between public and private partners. Ultimately, international policy collaboration was considered as a means of protecting the interests of public health systems faced with increasing involvement from private technology companies. Just as policy developments should consider all stages of the AI life cycle, so too must policy makers consider all potential actors.<br><br>Business and Use Case Development: National governments and international consortia are responsible for clearly outlining the needs of the global, national, and local health care systems that could derive maximum benefits from AI-driven technologies (...) The interviewees advocated setting a vision for using AI-driven technologies in the health system at a national rather than state or provincial level, with opportunities for local interpretation and implementation.<br><br>Deployment: Collaboration and multidisciplinary working by policy makers, technologists, health care professionals, and academics are needed to ensure appropriate expertise throughout the AI life cycle, especially during deployment of the technology into practice. Supporting research and implementation collaborations at a

	local level (eg, within a specific hospital or city) would create local showcase projects of AI research translated into practice. The design and execution of AI-driven technology trials require multidisciplinary approaches to assess clinical efficacy, comparative benefit and cost-effectiveness, and impact on clinical pathways and practice. (…) The participants strongly favored active stakeholder involvement in the development of governance mechanisms.
WHO, 2021[3]	Governments: Data governance – (…) governments might wish to define when consent can be waived in the public interest. This is already permissible under data protection laws if it is strictly necessary and proportionate to achievement of a legitimate aim. (…) This could include situations in which there are clear public health benefits of using data that would otherwise be unavailable because too many individuals have opted out of sharing such data.<br>Have clear data protection laws and regulations for the use of health data and protecting individual rights, including the right to meaningful informed consent.<br>Establish independent data protection authorities with adequate power and resources to monitor and enforce the rules and regulations in data protection laws.<br>Require entities that seek to use health data to be transparent about the scope of the intended use of the data.<br>Ensure that the growing provision of health-related services through online platforms that are not associated with the formal health-care system is identified, regulated (including standards of privacy protection guaranteed within health-care systems) and avoided for areas of health care in which the safety and care of patients cannot be guaranteed. Governments should ensure that patients who use such services also have access to appropriate formal health-care services when required.<br>Consider adopting models of co-regulation with the private sector to understand an AI technology, without limiting independent regulatory oversight. Governments should also consider building their internal capacity to effectively regulate companies that deploy AI technologies and improve the transparency of a company's relevant operations.<br>Consider establishing dedicated teams to conduct objective peer reviews of software and system implementation by examining safety and quality or general system functionality (fitness for purpose) without requiring review or approval of a code.<br>Consider which aspects of health-care delivery, financing, services and access could be supplied by companies, how to hold them accountable and which aspects should remain the obligation of governments.

	Assessing whether AI is necessary and appropriate for use in the public sector. As for any use of AI by health professionals, governments must assess whether an AI technology is necessary and appropriate for the intended use and can be used according to its laws.<br>(…) there is growing expectation that governments will be transparent about their use of AI, including whether they are investing in AI, engaged in partnerships with companies or developing AI independently in state-owned enterprises or government agencies. It is also expected that governments will be transparent about any harm caused by use of AI and the measures taken to redress any harm.<br>Conduct transparent, inclusive impact assessments before selecting or using any AI technology for the health sector and regularly during deployment and use (…) (ethics, human rights, safety, and data protection impact assessments).<br>Transparency in regulatory procedures and in interoperability should be enhanced and should be fostered by governments as deemed appropriate<br>Define legal and ethical standards for procurement of AI technologies and require public and private health-care providers to integrate those standards into their procurement practices.<br>Be transparent about the use of AI for health, including investment in use, partnerships with companies and development of AI in state-owned enterprises or government agencies, and should also be transparent about any harm caused by use of AI.<br>Governments should develop and implement ethical, legally compliant principles for the collection, storage and use of data in the health sector that are consistent with internationally recognized data protection principles. In particular, governments should take steps to avoid risks of bias in data that are collected and used for development and deployment of AI in the public sector.<br>Ensure that any use of AI to facilitate access to health care is inclusive, such that uses of AI do not exacerbate existing health and social inequities or create new ones.<br>Introduce and enforce regulatory standards for new AI technologies to promote responsible innovation and to avoid the use of harmful, insecure or dangerous AI technologies for health.

	Support global governance of AI for health to ensure that the development and diffusion of AI technologies is in accordance with the full spectrum of ethical norms, human rights protection and legal obligations.<br>Governments and national health authorities should ensure that decisions about introducing an AI system for health care and other purposes are taken not only by civil servants and companies but with the democratic participation of a wide range of stakeholders and in response to needs identified by the public health sector and patients. They should include representatives of public interest groups and leaders of marginalized groups, who are often not considered in making such decisions.<br>Governments should enact laws and policies that require government agencies and companies to conduct impact assessments of AI technologies, which should address ethics, human rights, safety and data protection, throughout the life-cycle of an AI system.<br>Regulatory agencies: Protection and oversight - Regulation could differ according to risk, such that those who are especially vulnerable, including people with mental illness, children and the elderly, are protected from misinformation and bad advice from health applications that exploit rather than assist such individuals. People living in resource-poor settings, in countries with inadequate resources to regulate and monitor adverse consequences of AI applications and with diseases that result in marginalization and discrimination, such as HIV/AIDS or tuberculosis, also require greater protection and oversight by regulatory agencies than users of applications for lifestyle or wellness.<br>Regulatory agencies should create incentives to encourage developers to identify and avoid biases.<br>Regulatory agencies in LMIC could consider either relying on regulatory approval of AI technologies in HIC or use of collaborative registration procedures to ensure that new technologies are appropriate for use.<br>Regulators should require that an AI system's performance be tested and sound evidence obtained from prospective testing in randomized trials and not merely from comparison of the system with existing datasets in a laboratory.<br>Regulators should require the transparency of certain aspects of an AI technology, while accounting for proprietary rights, to improve oversight and assurance of safety and efficacy. This may include an AI technology's source code, data inputs and analytical approach.<br>Regulators should provide incentives to developers to identify, monitor and address relevant safety- and human rights-related concerns during product design and development and should integrate relevant guidelines into

	precertification programmes. Regulators should also mandate or conduct robust marketing surveillance to identify biases.<br><br>Indigenous communities and other marginalized groups: Mechanisms for community oversight of data should be supported. These include data collectives and establishment of data sovereignty by indigenous communities and other marginalized groups.<br><br>Data hubs: should meet the highest standards of informed consent if their data might be used by the private or public sector, should be transparent in their agreements with companies and should ensure that the outcomes of data collaboration provide the widest possible public benefit.<br><br>Companies: must adhere to national and international laws and regulations on the development, commercialization and use of AI for health systems, including legally enforceable human rights and ethical obligations, data protection laws, measures to ensure appropriate informed consent and privacy.<br><br>Companies should invest in measures to improve the design, oversight, reliability and self-regulation of their products. Companies should also consider licensing or certification requirements for developers of "high-risk" AI, including AI for health.<br><br>Companies should ensure the greatest possible transparency in their internal policies and practices that implicate their legal, ethical and human rights obligations as established under the UN Guiding Principles on Business and Human Rights. They should be transparent about how those ethical principles are implemented in practice, including the outcomes of any actions taken to address violations of such principles.<br><br>Companies and developers should conduct impact assessments as per the UN Guiding Principles on Business and Human Rights, even if governments have not mandated them.<br><br>WHO: WHO should ensure clear understanding of which types of rights will apply to the use of health data and the ownership, control, sharing and use of algorithms and AI technologies for health<br><br>WHO should work in a coordinated manner with appropriate intergovernmental organizations to identify and formulate laws, policies and best practices for ethical development, deployment and use of AI technologies for health.<br><br>WHO should consider issuing model legislation to be used as a reference for governments that wish to build an appropriate legal framework for the use of AI for health.

	WHO, Intergovernmental organizations, major global health bodies: First, technical advice from and the engagement of WHO and other intergovernmental organizations such as the Council of Europe, OECD and UNESCO and respect for ethical principles and human rights standards can ensure that companies and governments both move towards common high standards. In the domain of global health, this will also require that major global health bodies, such as WHO, the Global Fund to Fight AIDS, Tuberculosis and Malaria, United Nations development agencies and foundations, agree on a common position about the risks associated with these technologies and clearly commit themselves to adherence to human rights and ethical standards as a core principle of all strategies and guidance.<br>(…) nongovernmental organizations and community groups: ensuring that human rights obligations and ethical principles are considered from the onset of decision-making and respected in practice and that governments and companies introduce appropriate safeguards to prevent and respond to any risks and swiftly redress any negative consequences of the use of AI.<br>Global health bodies: Global health bodies such as WHO, Gavi, the Vaccines Alliance, the Global Fund to Fight AIDS, Tuberculosis and Malaria, Unitaid and major foundations should commit themselves to ensuring that adherence to human rights obligations, legal safeguards and ethical standards is a core obligation of all strategies and guidance.<br>International agencies, such as the Council of Europe, OECD, UNESCO and WHO, should develop a common plan to address the ethical challenges and the opportunities of using AI for health, for example through the United Nations Interagency Committee on Bioethics.<br>International agencies (and professional societies) should ensure that their clinical guidelines keep pace with the rapid introduction of AI technologies, accounting for the evolution of AI technologies by continuous learning.<br>WHO should support national regulatory agencies in assessing AI technologies for health.<br>WHO should support countries in evaluating the liability regimes that have been introduced for the use of AI technologies for health and how such regimes should be adapted to different health-care systems and country contexts.<br>WHO and partner agencies should seek to establish international norms and legal standards to ensure national accountability to protect patients from medical errors

	Civil society: participate in the design and use of AI technologies for health as early as possible in their conceptualization.<br><br>Designers, developers, and users: Responsiveness requires that designers, developers and users continuously, systematically and transparently assess AI applications during actual use. They should determine whether AI responds adequately and appropriately and according to communicated, legitimate expectations and requirements<br><br>Designers and other stakeholders should ensure that AI systems are designed to perform well-defined tasks with the accuracy and reliability necessary to improve the capacity of health systems and advance patient interests. Designers and other stakeholders should also be able to predict and understand potential secondary outcomes.<br><br>Designers should ensure that stakeholders have sufficient understanding of the task that an AI system is designed to perform, the conditions necessary to ensure that it can perform that task safely and effectively and conditions that might degrade system performance.
Reddy et al, 2020[4]	We recommend a data governance panel constituted by the AI developers that includes patient and target group representatives, clinical experts, and people with relevant AI, ethical, and legal expertise. The panel would review datasets used for training AI to ensure the data is representative and sufficient to inform requisite model outcomes. (…) The panel would work to achieve a clearly articulated data collection and utilization strategy that will guide documentation, workflow, a review of influencing factors and monitoring standards. (…) Normative standards for the application of AI in health care should be developed by governmental bodies and healthcare institutions as part of governance. (...)<br><br>The FDA process could be similarly adopted by respective regulatory agencies across the world. In countries that do not have established regulatory processes for evaluation and monitoring of SaMD, there is a role for international bodies (eg, the World Health Organization) to guide and support relevant countries to adopt appropriate processes to regulate SaMD. (...) Professional medical bodies have a role in issuing clinical guidelines regarding where AI applications can be utilized in the diagnosis and treatment process (...). Such guidelines would increase not only the confidence of physicians using AI, but also their trust in AI applications. It would also respect the autonomy of patients.<br><br>Health services reviewing AI products in the market, assessing them for their suitability in their healthcare delivery and establishing relevant policies and procedures to allow for incorporation of AI software in clinical care. (...) To support the integration and governance, we recommend that governance be provided by a clinical governance

	committee formulated with specific skills and experience to oversee the introduction and deployment of AI models in clinical care. An appropriate governance committee should include clinicians, managers, patient group representatives, and technical and ethics experts so that appropriate deliberations are held about the efficacy and effectiveness of the AI models in addition to oversight of privacy, safety, quality, and ethical factors. Such a governance body should also ensure that an appropriately resourced team and plan is in place to monitor for data drift, input–output variation, unexpected outcomes, data reidentification risk, and clinical practice impacts. These efforts should be reported back up to the clinical owner and it should be the responsibility of the governance to enforce.
Jaremko et al, 2019[5]	CAR to work together with other stakeholders such as provincial Ministries of Health and the Canadian Medical Protection Association to develop guidelines for appropriate deployment of AI assistive tools in hospital departments and radiology groups, seeking to minimize potential harm and institutional liability for malpractice in case of medical error involving AI.<br>Radiologists using AI should be aware of its limitations, use AI appropriately within algorithms of care, and not allow AI to replace human expert judgment.
Parker et al, 2024[6]	AI governance bodies: should have an open line of communication with leadership and decision-makers. AI governance bodies must be empowered within their organizations to assess AI tools within their purview, and those decision-makers need to take recommendations seriously. We also found that governance teams were often multidisciplinary to ensure that governance is a holistic process.<br>While methods can vary, AI governance bodies must have a system in place to identify AI tools under consideration for implementation at their organization, whether these tools are commercially available or were developed internally. Once identified, AI governance teams collect information on the AI tool that will be used (...) to maintain an inventory of AI tools assessed or implemented in the health system.<br>Design with users: The patient, HCPs and relevant stakeholders need to be involved in the design of AI-driven technologies from the start to ensure the resultant product or service meets clinical, user and professional needs and complements existing workflows and experiences. Demonstrable benefit: Countries should focus on engaging and generating trust with the public, HCPs, industry, and other stakeholders through delivering AI-driven technologies that are concentrated on meeting a need(s) within the health system. Doing so moves the conversation about the public acceptability of AI away from the theoretical to one of showing the benefit and value AI-driven technologies

	bring to the health system. (...) The diverse strategies employed by different health systems highlight the continued need for flexibility in governance approaches, factoring in health systems' specific considerations around resources and processes. However, the number of commonalities found when exploring the different governance processes suggests that health systems should make use of published frameworks and guidance as they create their own processes.<br><br>Decision making authority: A critical piece of governance design is identifying who has the ultimate decision-making authority on whether a given AI tool will be implemented or decommissioned. This authority varied by organization. Some gave this authority to the person who allocated budget funds for the AI tool, and the review process is meant to guide this decision. Other health systems favored a more centralized decision process, where the review team or a larger governance group make the final decision. Still other health systems placed some or all decision-making with executive leadership, who rely on recommendations from the review process. This can ensure AI tool selection is consistent with the overall AI standards and strategy.<br><br>Health systems: Health systems must ensure they are aware when AI tools are being considered in order to bring them into the governance process. There were a variety of strategies for this, including general informational campaigns, directed conversations with individuals involved in purchase decisions, and training with internal AI developers on how and when to engage with the governance committee.<br><br>Health system leaders: should prioritize AI governance now and seek learning from early adopters, assessing what is the right-sized approach for their specific circumstances. However, health systems that have already built governance systems or have significant expertise in AI (…) also have a role in democratizing AI across settings. These systems should share documentation on how their governance systems work, including tools such as registration/information intake forms and surveillance procedures, and consider partnering with other health systems, especially those with fewer resources. At the same time, all health systems need to work to diffuse knowledge about AI and responsible AI implementation throughout their workforce. (...) health systems can create peer-to-peer learning spaces to educate, share and support each other implementing best practices in AI governance.<br><br>Federal and state governments: At the state level, Colorado recently passed a law that will require deployers of AI (…) to implement a risk-management system, conduct impact assessments, do annual reviews, and report any discoveries of algorithmic discrimination. (…). The government can also create positive incentives around governance such as safe harbors for health system deployers that employ best practices to reduce some of the risk

	in deploying AI tools, such as liability. The government can also prioritize funding research to simplify governance and make it more efficient. This may include creating research funding priorities around governance best practices, maturity models, and infrastructure to make monitoring for performance draft and bias and general surveillance more efficient. Government could also fund development of open-source tools such as inventory systems and testing tools to make the governance process less burdensome. (…) the government could help build a registry of AI tools similar to ClinicalTrials.gov that would also have a federated component linking to health system assessments of that tool. (…). Finally, the government could consider establishing and funding Health AI Technical Centers of Excellence to provide training modules for staffing governance teams and act as an expert resource for under-resourced health systems, as well as general workforce development around AI literacy.<br><br>Developers: Developers and health systems should be working together to create standardized checklists of information for different types of AI tools, to set appropriate expectations and increase transparency. This would also allow developers to create a standard information disclosure form that could be shared with governance teams that could reduce the amount of back-and-forth communication between developers and governance teams, increasing governance efficiencies.<br><br>Developers should also work to foster collaboration and trust with health systems. Aligning on expectations early and improving understanding of health system legal compliance requirements would be helpful<br><br>Developers can also create tools to facilitate local governance. One company recently announced that they would provide kits to simplify local tuning and testing of their products. This aligns with the previously mentioned FDA transparency principles stating that it would be helpful for developers to provide information on "how to conduct local site-specific acceptance testing or validation" and "plans for ongoing performance monitoring." Another company we spoke with described tools that would be able to automate monitoring for performance drift.<br><br>Clinical societies, public-private partnerships, and standards group: These entities should focus on creating guidance in this space (validation of health AI tools).<br><br>Medical, nursing, and other clinical professional schools and training programs: Should develop curricula on best practices in assessment and using AI tools while clinical societies and other organizations should establish continuing education courses on responsible governance and use of AI.

AAAiH, 2023[7]	Government: To better coordinate and harmonise the responsibilities and activities of those entities responsible for oversight of AI safety, effectiveness, and ethical and security risks; establish a National AI in Healthcare Council; Co-design and collaboratively implement a nationally accessible program for digital health literacy to inform the public of AI's benefits, risks and safe use, and increase public trust and confidence in AI; Assist professional bodies in accessing expertise and prior models to support the development of profession-specific codes of practice for the responsible use of AI; Work together with Aboriginal and Torres Strait Islander communities to develop a mechanism that collates health-related data for use in AI in a culturally safe and trusted manner within their control, in line with principles of Indigenous Data Sovereignty; Develop mechanisms to provide industry with ethical and consent-based access to clinical data to support AI development and leverage existing national biomedical data repositories; Support the development of a National AI Capability Centre in Healthcare (NAICCH) to assist industry (and SMEs in particular) to bring products to market; Identify emerging AI markets and opportunities, and quantify the economic costs and benefits of AI in healthcare (including climate risks and benefits), and indicators of effective use of AI in national health priority areas (e.g. ageing, disability, mental health, Indigenous health, rural and remote health); Ensure professional codes of conduct and training; emphasise the role of clinicians in educating patients about the responsible use of AI; Develop national clinical AI procurement guidelines in partnership with the jurisdictions, health services and industry; Provide significant targeted support for healthcare AI research that builds sovereign capability and can translate to improved priority health services and support for industry; Provide support and incentives for local industry (and SMEs in particular).<br><br>National AI Capability Centre in Healthcare: Assist professional bodies in accessing expertise and prior models to support the development of profession-specific codes of practice for the responsible use of AI; The NAICCH would focus on the many unique aspects of AI in healthcare and should link with other relevant organisations such as CSIRO's National AI Centre to prevent overlap in roles; provide access to healthcare-specific technical consultancy services, guidance to industry on best practice in procurement and regulatory compliance, pathways to reimbursement, software licensing, and climate risk mitigation.<br><br>Royal Australian and New Zealand College of Radiologists: The Royal Australian and New Zealand College of Radiologists has published Standards of Practice for Artificial Intelligence and other Colleges are in the process of doing so but may lack the necessary expertise in-house.<br><br>Australian Medical Council and other national Board delegated bodies: developing or updating digital health standards for practitioners and education providers.

	Therapeutic Goods Administration: legal responsibility to regulate software-based medical devices including those embedded with AI.<br>Australian Commission on Safety and Quality in Health Care (ACSQHC): oversee quality of care standards that will need to embrace AI.
Solaiman 2025[8]	Examples of the role of manufacturers as stated in the Saudi Food & Drug Authority's Guidance on Artificial Intelligence and Machine Learning technologies based Medical Devices ("MDS-G010"):<br>• The manufacturer should assess whether the promised medical benefit is achieved is consistent with the state of the art.<br>• Manufacturers should provide assurance that metrics of effectiveness and safety include outcomes that are meaningful to patients and clinical outcome, i.e. measures of improvement in patient outcomes, clinical process or time efficiency, measures of acceptable unintended consequences, and absence of harm to patients.<br>• The manufacturer should generate evidence on device performance that can be generalized to the entire intended population, demonstrating that performance will not deteriorate across populations and sites.<br>• The effects of AI/ML-based medical devices should be evaluated in clinically relevant conditions, i.e. this requires integration into the existing clinical workflow<br>• Manufacturers in their study design should consider proactively the effects that their studies may have on healthcare organizations and potentially explore the possibility of prospective real-world studies in order to minimize selection bias, have more control over variables and data collection, and examine multiple outcomes.
Arnaout et al, 2024[9]	Leadership and Sponsorship from each health authority: Ensures standardization of processes and a provincial coordinated approach, prevents duplication of efforts. Allows equitable access to AI tools across BC.<br>Data Scientists and Engineers: Ensures data quality and accuracy, as well as collect, clean, and organize the large volumes of healthcare data required to continuously train and evaluate AI tools.<br>Patients: as end-users can provide valuable perspectives and insights into their needs, preferences, concerns, and experiences, which can inform the implementation of AI solutions.

	Diverse Partner Involvement: Incorporating diverse perspectives ensures a responsible, safe, and comprehensive approach to AI tool deployment in healthcare across BC.<br><br>Ethicists: To ensure human centricity of AI tools and consider ethical principles that underpin the potentially harmful effects of AI, as well as the use cases in which systems may be applied.<br><br>Medico-Legal and Risk Management: Addressing risks such as patient harm, bias, discrimination, and privacy infringement, while ensuring regulatory compliance, evaluation, and building public trust.
Whittaker et al, 2023[10]	The newly established Artificial Intelligence Governance Group (AIGG) agreed on one initial over-arching question with further considerations in eight domains.<br><br>Ensuring that there is clear public communication will, in part, be the responsibility of the AIGG as well as the service intending to develop or implement the models.<br><br>Consumer perspectives are prioritised throughout the process to ensure our population would be comfortable with the use of AI in the context proposed. (...). The inclusion of Māori perspectives in the AIGG also encourages Te Tiriti based arrangements, ensuring acceptability and accountability to Māori with a commitment to equitable benefits through a sustained focus on mana (mutual respect). (...) Clinical perspectives reflect the involvement of clinicians in the concept and development phases, their comfort with the evidence of accuracy, and how easily the AI can fit within existing clinical workflows. Without an understanding of how local health services currently work, it is entirely possible for new developments to completely miss the mark in assisting them, even as newer ways of working emerge. (...). Technical guidance is provided by AI development expertise on the AIGG that is specific to the local IT context with an understanding of both its data storage and presentation systems. Technical guidance may also involve national or local standards, cyber security guidance and approval from local security officers as needed.
Carter et al, 2024[11]	There must be an independent decision-making body to manage the charter. We recommend the board chair is independent of the health system and investors to avoid bias.<br><br>- Individual clinicians: understanding and evaluating AI as used in health care, including its shortcomings, and ensuring that training data are relevant to local people;<br><br>- Clinical training and accreditation bodies: ensuring that clinicians are knowledgeable about the use and limits of AI systems;

	- Patients' representatives: advocating patients' rights, the provision of quality information to patients, and standards for AI use, as well as holding decision makers to account;<br>- Health care organisations and service providers: auditing AI systems for integrity, performance, and bias in local populations before procurement, managing conflicts of interest, considering the use of open source software, ensuring the ongoing monitoring of overall health system performance;<br>- researchers and evaluators: auditing datasets for representativeness, rigorously and independently evaluating AI system performance in clinical care, and embedding ongoing monitoring and feedback; and<br>- Health departments and agencies: building public understanding of health care AI and incorporating public voices into decision making about AI in health care.
Liao et al, 2022[12]	Clinical domain: patients, clinicians and other front-line users of the AI solution (…). The goal of governance for this domain is maintaining patient safety, as well as securing clinician acceptance and adoption.<br>Operational domain: systems-level components that are part the care delivery mechanism. This group includes the stakeholders that represent clinical operations, information services and informatics. (...) The goal of the governance for this domain is complementary oversight that is compatible with the routine operating model of the health system.<br>Leadership domain: those who manage the strategic direction of the health system, hold key decision rights, and govern the resources. (...) The goal of the governance for this domain is endorsement by senior leadership in health operations. (...) Workgroups were established in an ad hoc fashion. The responsibility of these workgroups was performing due diligence and providing detailed scrutiny of the AI solutions to establish the necessary validity both clinically, technically, and ethically. (...) The designation of these workgroups evolved along with the AI maturity of the organization, beginning with "algorithm workgroups", then to "algorithm committees", with a current designation of "algorithm sub-committees".<br>We created an institutional level steering committee which would provide a front door and maintain oversight of all models while retaining individual workgroups for more detailed governance. This "Clinical AI and Predictive Analytics Committee" is multidisciplinary and included a superset of the same disciplines that comprised the use-case specific algorithm workgroups. The committee functions as a front door for the evaluation and vetting of predictive solutions prior to implementation, and for new models it commissions and oversees workgroups. The committee reports up to existing clinical and informatics leadership structures in the university and health system

	and provides visibility on all clinical uses of AI to these groups. The institutional-level committee defines and establishes definitions of key terms such as "predictive model" as well as guiding principles. (…) IT professionals have a prominent role within our governance structure, AI application deployments are viewed as clinical projects analogous to other clinical initiatives in the hospital. (...) To address the equitable and ethical use of AI, the membership of our institutional committee includes ethics expertise, including a prominent faculty member from the Law School, and staff from our office for Diversity, Equity, and Inclusion, and we maintain a line of communication with our medical bio-ethicists.
Bedoya et al, 2022[13]	Development team: define and document the clinical and model performance requirements (…) The development team also partners with information technologists and implementation science experts to refine the UI and workflow. (...) The development team could engage with the ABCDS governance to: (1) review current progress; (2) review model performance characteristics and/or application to the outcome of interest; (3) assess adherence to clinical decision support and technological best practices; and (4) assess readiness for progression to the next lifecycle phase.<br><br>The Machine Intelligence Tracking Platform for Algorithms (MITRA) captures facts related to an algorithm, including its clinical and operational stakeholders, indication, methodology, and past/current versions, among other metadata.<br><br>Three subcommittees comprising specialists with expertise relevant to each checkpoint's focus. (...) The evaluation subcommittee reviews the implementation plan, focusing on workflow impact and UI design. Clinical and regulatory experts may offer guidance on how the final UI and workflow design could affect whether the ABCDS tool is characterized as SaMD. (...) The regulatory framework for the model and model classification (eg, as SaMD) are assessed by specialists with expertise in FDA medical device regulatory affairs within our School of Medicine. (...) Monitoring criteria established during G2, including model performance, stability, adoption, clinical outcome, and fairness and equity metrics are reviewed. Justification by clinical and business owners are provided for ongoing deployment and use.<br><br>An executive-level committee that provides institution-wide oversight and governance. At our institution, this committee is cochaired by the Duke Health System's chief health information officer and the School of Medicine's vice dean for data science.<br><br>It also includes the chairs of the 3 subcommittees that scrutinize models at their respective checkpoints (Figure 1), as well as representatives of health system nursing and innovation. This committee defines the ABCDS lifecycle

	and checkpoints as well as cataloguing, review, and approval of all model deployments as they progress through their lifecycles. The work of this committee and the respective subcommittees is organized and managed by an experienced program leader with expertise in the health IT product lifecycle.
Hassan et al, 2025[14]	Not stated
Economou-zavlanos et al, 2024[15]	Development teams must notify the OC of intentions to deploy any algorithmic technology (whether developed locally or sourced from a vendor) that could affect patient care or clinical operations. (…) The development team must describe how the technology's design and proposed workflow integration may influence its potential to be considered Software as a Medical Device (SaMD) by the Food and Drug Administration (FDA).<br>Before proceeding to each new phase of development or deployment, teams must provide evidence to demonstrate alignment with the 5 principles.<br>A subset of OC members designated as the Review Committee (RC) then evaluates results from completed phases, assesses readiness for subsequent phases, and provides structured feedback in a short survey (Exhibit S3); subject matter experts are consulted as needed. In collaboration with institutional regulatory advisors, the RC also provides guidance on appropriate regulatory pathways and, where applicable, FDA engagement.
Daye et al, 2022[16]	An imaging AI governing body has the responsibilities of defining the purposes, priorities, strategies, and scope of the group; establishing a framework for operation; and linking those to the organizational mission, values, vision, and strategy. AI governance structures provide mechanisms to decide which tools should be deployed locally and how to best allocate institutional and/or departmental resources to support the clinical implementation of the most valuable and highest-impact applications to improve patient care. Governance committees can establish a robust process to score and evaluate AI-based solutions objectively. (…) Free-flowing, multidirectional communication should occur between the imaging AI governing body, the broader organization that empowers it, the system-wide informatics governing bodies, and the end users of each AI tool. Incorporating end users into the governance structure is of utmost importance to consider their needs and concerns about an algorithm and to include it into the decision-making process.

	Issues that might be less important for academic practices, such as which entity pays to install and maintain the AI models, will be important in community hospital settings, and radiologists will need to develop win-win scenarios with AI models that are valuable both to the radiologists and to the health system. (...) radiologists must have a role in the decision-making process before these models are used. If a health system bears the financial burden for AI, radiologists must develop the value proposition for each model. If models are seen as only improving radiologist efficiency or accuracy, then the radiology group may be asked to bear some or all of the financial cost. (…) The governance team will have to expand to include diverse experts who can review evidence, perform utility analysis, estimate risk, assess technical and clinical readiness, and predict economic effects. (…) Before implementation, the governing committee should establish safeguards to prevent patient harm, especially in high-risk scenarios, such as screening applications in otherwise healthy populations or for tools that make drug or treatment recommendations. Although the regulation of AI is in its early stages, a legal representative should be included in every AI governance committee. For every algorithm, the risk for liability and malpractice should be discussed.
Kim et al, 2023[17]	Frontline workers to provide an authentic sense of context and help to characterize the problem.<br>End users in the early stage of problem formulation to anticipate and mitigate these potential barriers to adoption. (…) Some organizations founded governance committees with enterprise-wide scope to prevent hasty procurement happening in silos (external forces might distract problem-led procurement). The participants reported that in making decisions to develop or adapt AI software, all affected parties from various disciplines, including AI specific committees and specialists in clinical, operational, and technical areas, must be aligned.<br>Participants often recommended a centralized group with dedicated resources be accountable for monitoring the AI software. They proposed that joint accountability between clinical, statistical, and technical providers would be ideal for continued use of the model.
Apfelbacher et al, 2024[18]	Before the implementation: An AI committee should be established, including experts in i. a. data protection, ethics, IT, chairman of the hospital board, medical informatics, and patient representative. This committee will provide advisory support, ensure close coordination between the departments involved, and have veto power to adjust or reject implementation of the AI application (...). Safety is ensured through feedback loops, emergency numbers, and a designated contact person at the manufacturer´s side for emergencies. Physicians are primarily responsible for medical errors, but the manufacturer is liable for system related problems (...).

	The application needs to be monitored and eventually adjustments to the patient cohort of the UKER may need to be made. These checks and adjustments should be carried out at regular intervals by independent audit organizations.
Kim et al, 2026[19]	Roles and responsibilities of the AI governance committee members:<br>Clinical and medical:<br>• Advise on clinical risks of use and operational value<br>• Ensure alignment to clinical operations priorities, workplan, feasibility, and viability<br>• Participate in evaluation design and implementation<br>• Ensure appropriate engagement and change management plans are in place<br>• Monitor system impacts and unintended consequences<br>Technical:<br>• Evaluate the proposed AI implementation by assessing model performance, UI/UX, and solution performance<br>• Ensure access and security parameters<br>• Create test environment and architecture for data flows<br>• Monitor system impacts and unintended consequences<br>Social:<br>• Advise on workflow<br>• Identify potential risks of implementation<br>• Assess impact of technology on work environment of the solution<br>• Establish feedback mechanisms from users and affected patients<br>Informatics:<br>• Advise on data quality and availability<br>• Understand the model and determine the fields required for model

	• Confirm data model for implementing and monitoring AI solution and facilitate integration into legacy systems<br>• Participate in evaluation design and implementation<br>Operational:<br>*For operating and implementing specific AI solutions:*<br>• Ensure strategic alignment<br>• Ensure appropriate engagement and change management plans are in place<br>• Ensure new clinical workflows are followed<br>• Monitor system impacts and unintended consequences<br>• Assess long range funding, financial opportunities, and impacts and ensure return on investment<br>*For operating the AI governance committee:*<br>• Develop communication plans for implementing AI governance process<br>• Source and maintain necessary documentations of AI solutions<br>• Ensure timely completion of governance activities and balance administrative burden with appropriate guardrails<br>• Monitor the effectiveness of AI governance<br>Ethics and legal:<br>• Ensure AI implementation follows internal policies, procedures, and governance<br>• Ensure AI implementation follows legal requirements and privacy laws<br>• Ensure AI implementation follows ethical principles<br>• Assess impact on privacy and security and develop risk management plan for AI implementation<br>• Assess regulatory compliance<br>• Monitor national technology policy and regulation landscape

This is a Multimedia Appendix to a full manuscript published in the J Med Internet Res. For full copyright and citation information see https://www.jmir.org/2026/1/e87448

Alami H, Pozelli Sabio R, Pérez EJ, Gagnon MP, Langlois L, Denis JL, Malas K, Rivard L, Salvodelli M, Ag Ahmed MA, Fortin JP
Artificial Intelligence Governance in Health Systems: Systematic Review of Frameworks and Integrative Model Proposal
J Med Internet Res 2026;28:e87448

References

1. World Health Organization. Ethics and Governance of Artificial Intelligence for Health: Guidance on Large Multi-Modal Models. World Health Organization; 2024; Available from: https://www.who.int/publications/i/item/9789240084759.
2. Morley J, Murphy L, Mishra A, Joshi I, Karpathakis K. Governing data and artificial intelligence for health care: developing an international understanding. JMIR Form Res. 2022 Jan;6(1):e31623. PMID: WOS:000854067700049. doi: 10.2196/31623.
3. World Health Organization. Ethics and governance of artificial intelligence for health: WHO guidance. World Health Organization; 2021 [Accessed 2025-01-11]; Available from: https://www.who.int/publications/i/item/9789240029200.
4. Reddy S, Allan S, Coghlan S, Cooper P. A governance model for the application of AI in health care. J Am Med Inform Assoc. 2020 Mar 1;27(3):491-7. PMID: 31682262. doi: 10.1093/jamia/ocz192.
5. Jaremko J, Azar M, Bromwich R, Lum A, Alicia Cheong L, Gibert M, et al. Canadian Association of Radiologists White Paper on ethical and legal issues related to artificial intelligence in radiology. Can Assoc Radiol J May 2019;70(2):107-18. doi: 10.1016/j.carj.2019.03.001.
6. Parker V, Economou-Zavlanos N, Silcox C. AI governance in health systems: aligning innovation, accountability and trust. Duke Health; 2024 [Accessed 2025-01-11]; Available from: https://healthaigovernance.duke.edu/news/white-paper-ai-governance-health-systems-aligning-innovation-accountability-and-trust.
7. AAAiH. A national policy roadmap for artificial intelligence in healthcare. AAAiH; 2023 [Accessed 2025-05-05]; Available from: https://aihealthalliance.org/wp-content/uploads/2023/11/AAAiH_NationalPolicyRoadmap_FINAL.pdf.
8. Solaiman B. From bench to bedside: governing health care artificial intelligence (AI) through a “true lifecycle approach”. American Journal of Law & Medicine. 2025;51(3-4):452-78. doi: 10.1017/amj.2025.10091.
9. Arnaout A, Gill P, Virani A, Flatt A, Prodan-Balla N, Byres D, et al. Shaping the future of healthcare in British Columbia: Establishing provincial clinical governance for responsible deployment of artificial intelligence tools. Healthc Manage Forum. 2024;37(5):320-8. doi: 10.1177/08404704241264819.

10. Whittaker R, Dobson R, Jin CK, Style R, Jayathissa P, Hiini K, et al. An example of governance for AI in health services from Aotearoa New Zealand. NPJ Digit Med. 2023;6(1). doi: 10.1038/s41746-023-00882-z.
11. Carter S, Aquino Y, Carolan L, Frost E, Degeling C, Rogers W, et al. How should artificial intelligence be used in Australian health care? Recommendations from a citizens' jury. Med J Aust. 2024;220(8):409-16. doi: 10.5694/mja2.52283.
12. Liao F, Adelaine S, Afshar M, Patterson B. Governance of clinical AI applications to facilitate safe and equitable deployment in a large health system: key elements and early successes. Front Digit Health. 2022;4:931439. PMID: 36093386. doi: 10.3389/fdgth.2022.931439.
13. Bedoya A, Economou-Zavlanos N, Goldstein B, Young A, Jelovsek J, O'Brien C, et al. A framework for the oversight and local deployment of safe and high-quality prediction models. J Am Med Inform Assoc. 2022;29(9):1631-36. doi: 10.1093/jamia/ocac078.
14. Hassan M, Borycki E, Kushniruk A. Artificial intelligence governance framework for healthcare. Healthc Manage Forum. 2025 Mar;38(2):125-30. PMID: 39470044. doi: 10.1177/08404704241291226.
15. Economou-Zavlanos N, Bessias S, Cary M, Bedoya A, Goldstein B, Jelovsek J, et al. Translating ethical and quality principles for the effective, safe and fair development, deployment and use of artificial intelligence technologies in healthcare. J Am Med Inform Assoc. 2024;31(3):705-13. doi: 10.1093/jamia/ocad221.
16. Daye D, Wiggins W, Lungren M, Alkasab T, Kottler N, Allen B, et al. Implementation of clinical artificial intelligence in radiology: who decides and how? Radiology. 2022 Dec;305(3):555-63. PMID: 35916673. doi: 10.1148/radiol.212151.
17. Kim J, Boag W, Gulamali F, Hasan A, Hogg H, Lifson M, et al. Organizational governance of emerging technologies: AI adoption in healthcare. Presented at: FAccT '23: Proceedings of the 2023 ACM Conference on Fairness, Accountability, and Transparency; Chicago, IL, USA: Association for Computing Machinery; Jun 12-15, 2023. p. 1396–417.
18. Apfelbacher T, Kocman SE, Prokosch HU, Christoph J. A governance framework for the implementation and operation of AI applications in a university hospital. Stud Health Technol Inform. 2024 Aug 22;316:776-80. PMID: 39176908. doi: 10.3233/SHTI240527.
19. Kim JY, Hasan A, Balu S, Sendak M. People process technology and operations framework for establishing AI governance in healthcare organizations. npj Digital Medicine. 2026;9(1). doi: 10.1038/s41746-026-02419-6.
